# Pro-resolving lipid mediators in diseases: exploring the molecular basis and clinical implication

**DOI:** 10.1186/s43556-025-00400-5

**Published:** 2026-01-05

**Authors:** Chaofu Li, Zimu Wang, Yukun Yang, Qiuyan Jiang, Yingying Jiang, Jun Xiao, Li Shen, Wei Wu, Chuanwei Li

**Affiliations:** 1 Department of Cardiology, Chongqing University Central Hospital (Chongqing Emergency Medical Center), College of Bioengineering, Chongqing University, Chongqing, 400014 China; 2 Department of Cardiology, Zhongshan Hospital, Fudan University, Shanghai Institute of Cardiovascular Diseases, Shanghai, 200032 China; 3 Department of Neurology, University Hospital Essen, University of Duisburg-Essen, 45147 Essen, Germany; 4 National Clinical Research Center for Interventional Medicine, Shanghai, 200032 China

**Keywords:** Pro-resolving lipid mediators, Inflammation resolution, Biosynthesis and receptor signaling, Immune regulation and tissue repair, Translational medicine

## Abstract

Inflammation resolution is now understood as an active and highly coordinated biological process rather than a passive decline in inflammatory signals. When this program fails, inflammation may become persistent and gradually shift to a chronic pathological state. Such unresolved inflammation is increasingly recognized as a core driver of cardiovascular disease, metabolic disorders, autoimmune pathologies, neurodegeneration, and tumor progression. Although conventional anti-inflammatory drugs can suppress inflammatory mediators, they do not restore immune balance or actively promote resolution, and long-term administration may disrupt host defense and tissue-repair processes. Pro-resolving lipid mediators (PRLMs), including resolvins, maresins, protectins, and lipoxins, represent a distinct class of bioactive metabolites derived from polyunsaturated fatty acids. Recent studies have demonstrated that PRLMs regulate inflammation through specialized pro-resolving programs, such as enhancing efferocytosis, modulating cytokine networks, guiding leukocyte trafficking, and promoting tissue regeneration via receptor-dependent signaling pathways. These findings highlight a conceptual shift in inflammation management from broadly inhibiting inflammation to restoring immune homeostasis. Despite encouraging progress, several challenges hinder clinical translation, including rapid metabolic inactivation, limited delivery strategies, and unresolved pharmacological parameters. In this review, we summarize the current advances in PRLM biosynthesis, signaling pathways, and biological functions across multiple disease contexts. We also discuss emerging therapeutic strategies, biomarker development, and knowledge gaps that require further investigation. PRLM research offers a promising framework for next-generation resolution-based therapeutic interventions.

## Introduction

Therapeutic strategies for chronic inflammatory diseases are undergoing a paradigm shift from broad immunosuppression to approaches that actively promote inflammation resolution [1]. Resolution-based therapies differ from traditional immunosuppressive methods in that they harness endogenous pathways that terminate inflammation while preserving essential host defense [2, 3]. Pro-resolving lipid mediators (PRLMs) play a central role in this process by coordinating the clearance of inflammatory stimuli and facilitating tissue repair [4]. Targeting PRLM-driven pathways has emerged as a promising therapeutic strategy for diverse non-resolving chronic inflammatory diseases (NCDs), including cardiovascular disease, neurodegenerative disorders, metabolic syndrome, autoimmune disorders, and cancer [3].

Recent discoveries have redefined the resolution of inflammation as an active, programmed process rather than a passive outcome [5]. This process is orchestrated by PRLMs, which are bioactive molecules derived from polyunsaturated fatty acids (PUFAs). During the early phases of metabolic, ischemic, or infectious injury, inflammatory responses are dominated by proinflammatory cytokines (e.g., IL-1β, IL-6, and TNF-α), chemokines (e.g., MCP-1), and ω−6 fatty acid–derived eicosanoids (e.g., prostaglandins and leukotrienes (LTs), which recruit polymorphonuclear leukocytes (PMNs) and facilitate pathogen clearance [6]. Once the injurious stimuli are eliminated, the immune system undergoes a “lipid mediator class switch,” suppressing proinflammatory mediator production and redirecting ω−3 fatty acids toward the biosynthesis of PRLMs, including resolvins (Rvs), protectins (PDs), and lipoxins (LXs), which actively terminate inflammation and restore tissue homeostasis [7].

Mechanistically, PRLMs act through G-protein–coupled receptors (GPCRs) and multiple downstream signaling pathways. They promote apoptosis and clearance of inflammatory cells, enhance macrophage phagocytosis, limit the excessive release of proinflammatory mediators, and coordinate immune cell lineage remodeling. Collectively, these functions drive tissue repair and restore functional integrity [8]. By contrast, inadequate PRLM production or persistent noxious stimuli disrupts the balance between proinflammatory and pro-resolving responses, fostering the transition from acute to chronic inflammation, perpetuating tissue damage, and accelerating disease progression [9].

**Fig. 1:**
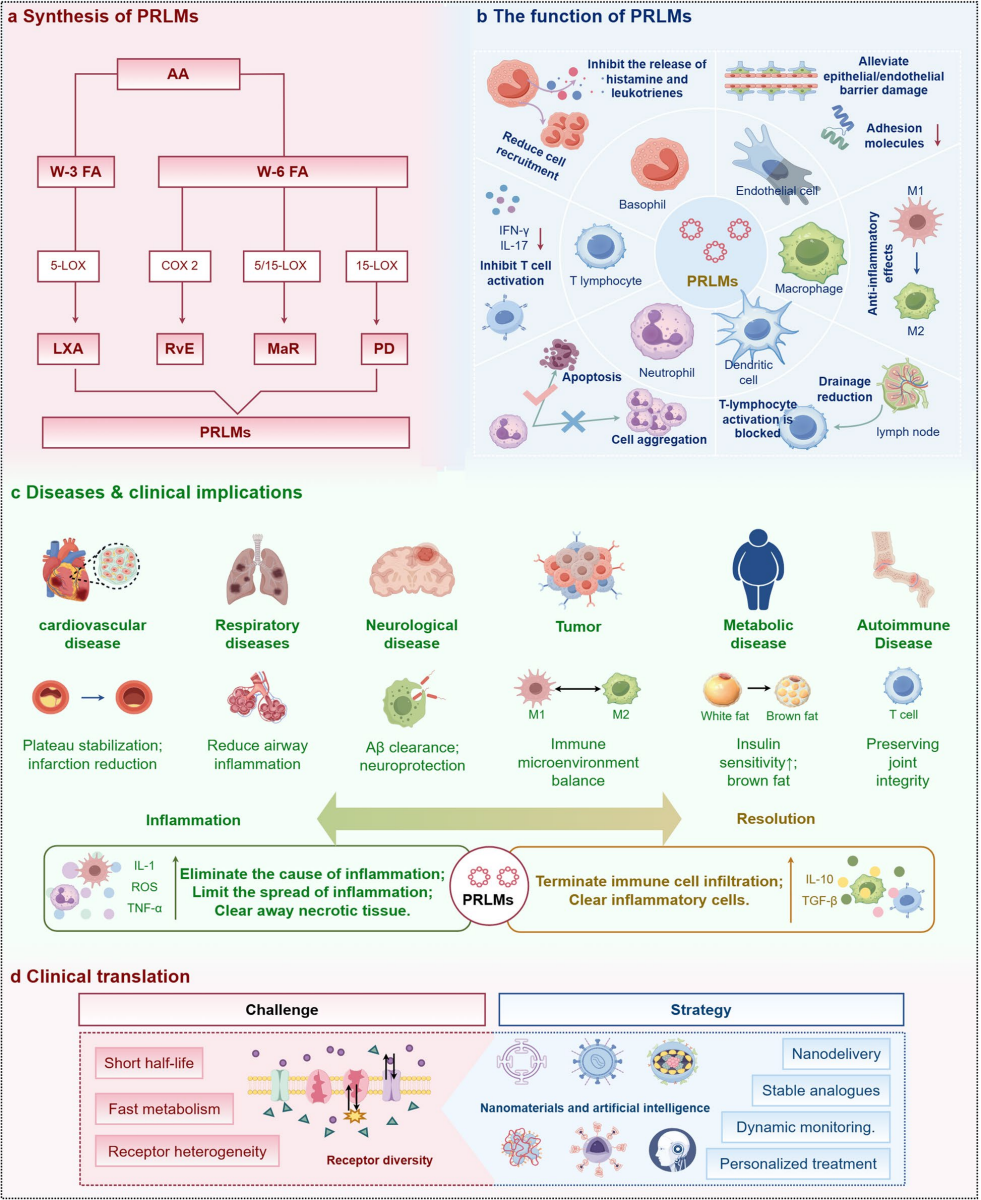
Pro-resolving lipid mediators (PRLMs): from biosynthesis to clinical translation (By Figdraw, License ID: ORIOA4c1d0). **a** Synthesis of PRLMs: Pro-resolving lipid mediators (PRLMs) are a distinct class of bioactive lipids generated from ω−3 and ω−6 fatty acids through LOX- and COX-dependent enzymatic pathways, giving rise to four major families—lipoxins (LX), resolvins (Rv), protectins (PD), and maresins (MaR). **b** The function of PRLMs: Rather than passively suppressing inflammation, PRLMs actively restore tissue homeostasis by limiting leukocyte infiltration, guiding macrophage polarization toward a reparative phenotype, and maintaining epithelial and endothelial barrier integrity. **c** Diseases and clinical implications: Impaired PRLMs biosynthesis or signaling is increasingly recognized as a common feature across cardiovascular, respiratory, neurological, metabolic, malignant diseases, and autoimmune disease highlighting their essential role in immune balance and resolution biology. **d** Clinial translation: Recent advances, including nanodelivery systems, stabilized structural analogues, and precision design aided by artificial intelligence, are helping to overcome limitations in stability and receptor heterogeneity, opening new avenues for their clinical translation

These mechanistic insights have positioned PRLMs and their receptor-mediated pathways as promising therapeutic targets for chronic inflammatory diseases [8]. Unlike conventional anti-inflammatory approaches, resolution-targeted interventions aim to restore immune balance and reduce the risks associated with long-term immunosuppression, highlighting their substantial translational potential [10]. This review summarizes the biosynthesis, signaling networks, and mechanisms of action of PRLMs, integrates recent preclinical evidence on chronic inflammatory diseases, and discusses both the therapeutic advantages and unresolved challenges. Together, these insights provide a conceptual foundation for developing next-generation precision therapies centered on the biology of inflammation resolution (Fig. 1).

## Molecular components and pathways in PRLM biology

**Fig. 2:**
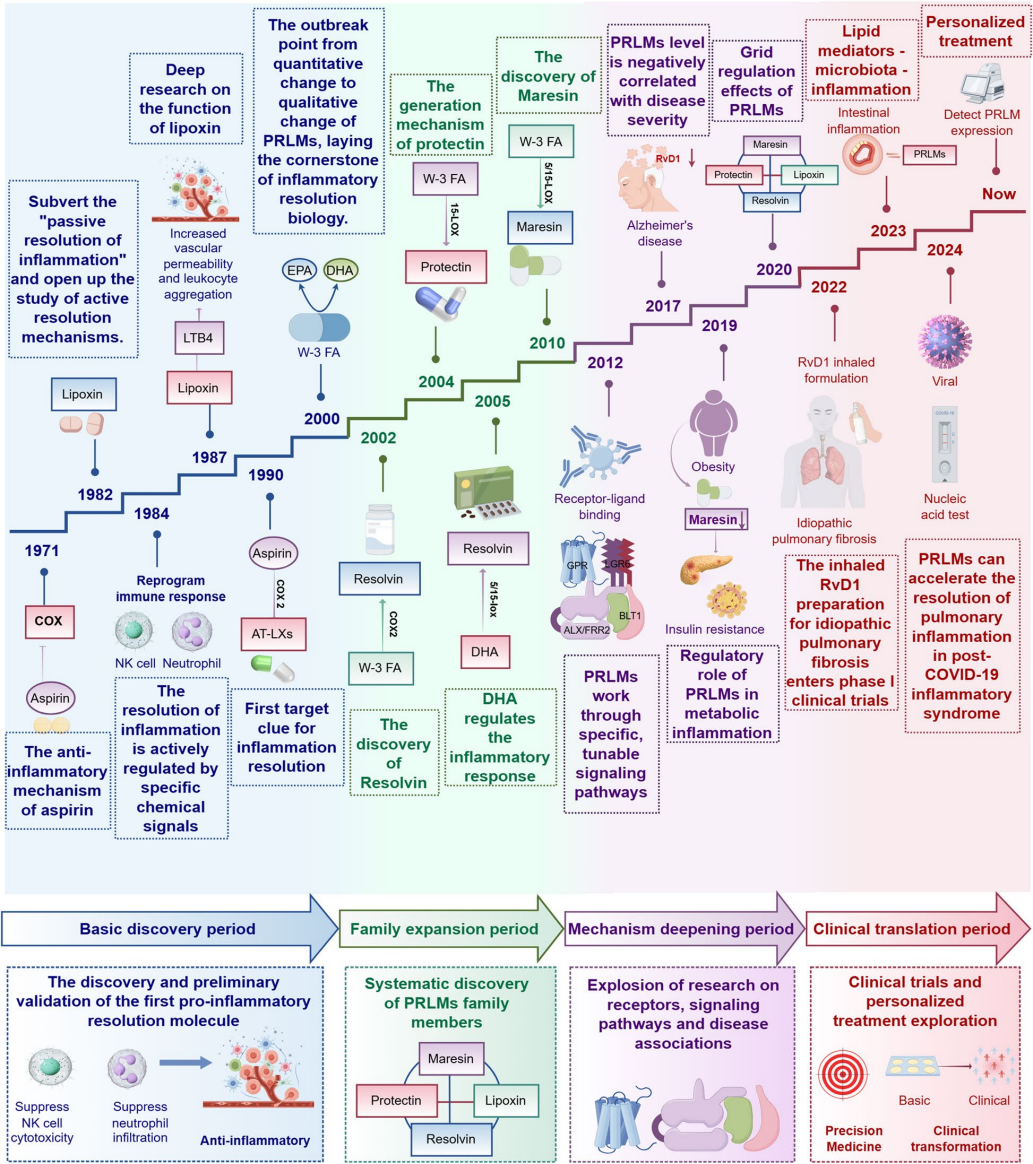
Timeline of key milestones in the discovery and translational development of PRLMs (By Figdraw, License ID: YAPUTa8433). The timeline summarizes major scientific advances from the identification of the anti-inflammatory mechanism of aspirin to the emergence of personalized PRLMs-based therapies. Four phases are highlighted: (1) the basic discovery period (1971–2000), defining active resolution of inflammation; (2) the family expansion period (2000–2010), with the discovery of lipoxins, resolvins, protectins, and maresins derived from ω−6 and ω−3 fatty acids; (3) the mechanistic deepening period (2010–2020), revealing receptor–ligand interactions and signaling networks; and (4) the clinical translation period (2020–present), featuring inhaled PRLMs formulations, lipidomic profiling, and personalized therapeutic strategies. Abbreviations: AA, arachidonic acid; COX, cyclooxygenase; CYP, cytochrome P450; DHA, docosahexaenoic acid; EPA, eicosapentaenoic acid; GPCR, G-protein-coupled receptor; LOX, lipoxygenase; MAPK, mitogen-activated protein kinase; mTOR, mechanistic target of rapamycin; NF-κB, nuclear factor-κB; PRLMs, pro-resolving lipid mediators

**Fig. 3:**
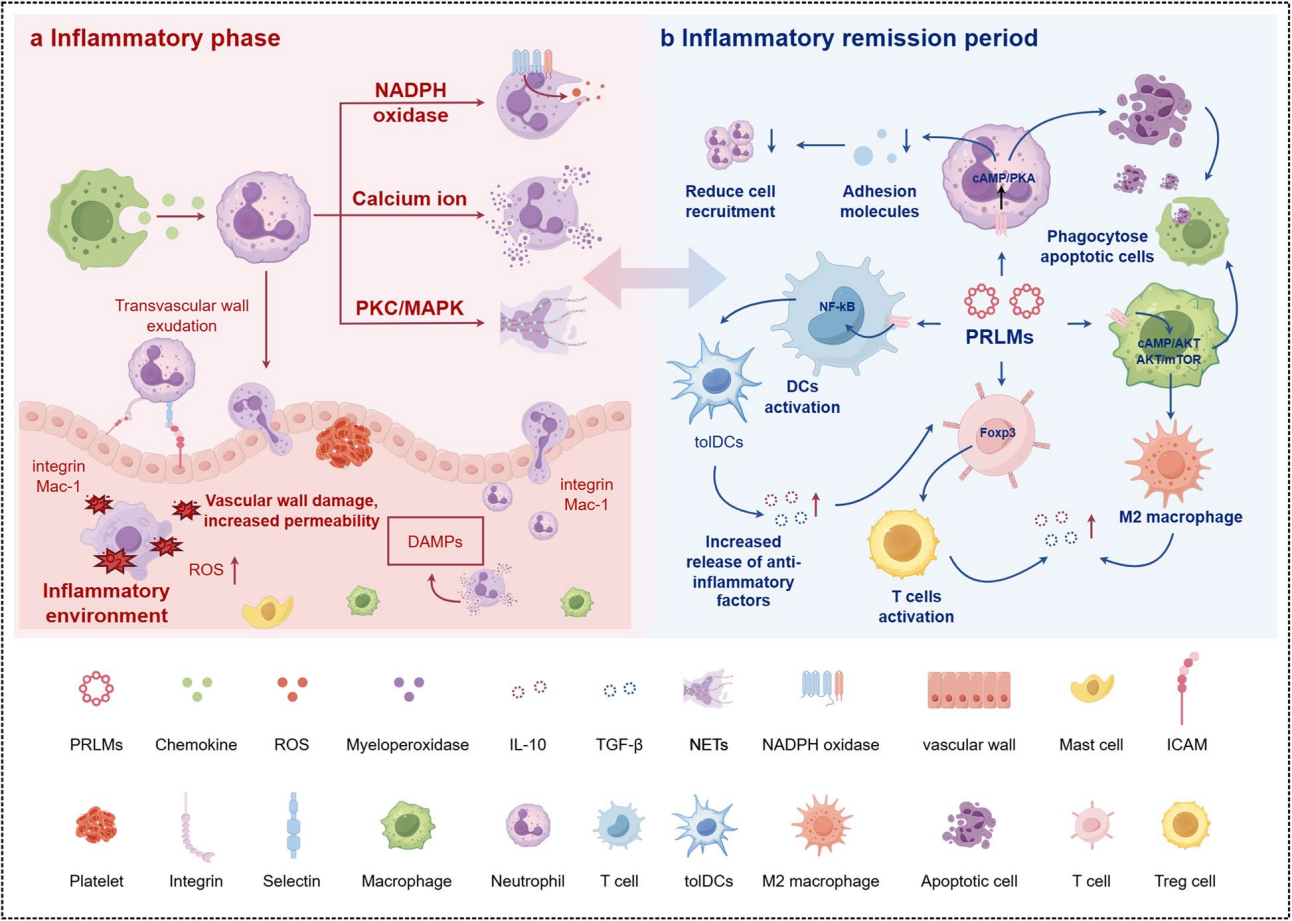
Inflammatory phase and pro-resolving actions of PRLMs during resolution (By Figdraw, License ID: OWTTPb979d). **a** Inflammatory phase: Tissue injury activates vascular endothelium and promotes neutrophil infiltration through Mac-1–integrin–mediated transendothelial migration. Activated neutrophils release reactive oxygen species (ROS), myeloperoxidase, and DAMPs, leading to vascular leakage and amplification of inflammation via NADPH oxidase, PKC, and MAPK signaling. **b** Resolution phase: Pro-resolving lipid mediators (PRLMs) inhibit leukocyte adhesion and NF-κB–driven inflammation, promote efferocytosis and M2 macrophage polarization through cAMP/PKA and AKT/mTOR pathways, and induce tolerogenic dendritic cells and Foxp3⁺ Treg activation. These processes enhance IL-10 and TGF-β release and restore immune homeostasis. Abbreviations: PRLMs, pro-resolving lipid mediators; ROS, reactive oxygen species; DAMPs, damage-associated molecular patterns; PKC, protein kinase C; MAPK, mitogen-activated protein kinase; NF-κB, nuclear factor kappa-B; PKA, protein kinase A; DCs, dendritic cells; Treg, regulatory T cell; ICAM, intercellular adhesion molecule

Since the identification of the first class of PRLMs in 1984, our understanding of their biosynthesis, receptors, and functions has advanced considerably [11]. The discovery of lactylated PRLMs in 2019 further expanded this research area (Fig. 2). Extensive studies have shown that PRLMs actively resolve inflammation and restore tissue homeostasis through coordinated actions, such as inhibiting neutrophil infiltration, promoting macrophage phagocytosis, and initiating tissue-repair processes. This challenges the previous view that PRLMs passively await inflammation resolution. However, when the synthesis or function of PRLMs is disrupted, the resolution process is impaired, leading to persistent inflammation, continued tissue damage, and progression from acute to chronic inflammation. Thus, PRLMs act not only as the “brakes” in the inflammatory process but also as “navigators” that guide the transition from inflammation to a healthy state. Research on PRLMs has opened new therapeutic avenues for chronic inflammatory diseases, shifting the focus from traditional “anti-inflammatory” strategies to “pro-resolution” therapies [5] (Fig. 3). Recently, advances in high-resolution mass spectrometry, lipidomics, and deep learning–based receptor–ligand prediction have enabled the discovery of novel PRLMs and their candidate receptors with unprecedented spatiotemporal resolution [11]. These tools have also provided new insights into the mechanistic roles of these compounds in the regulation of inflammation and disease pathogenesis. Together, these technological innovations not only facilitate the mapping of PRLMs’ functional networks but also create opportunities for developing resolution-based therapeutic strategies.

### Key biosynthetic enzymes and their regulatory checkpoints

**Fig. 4:**
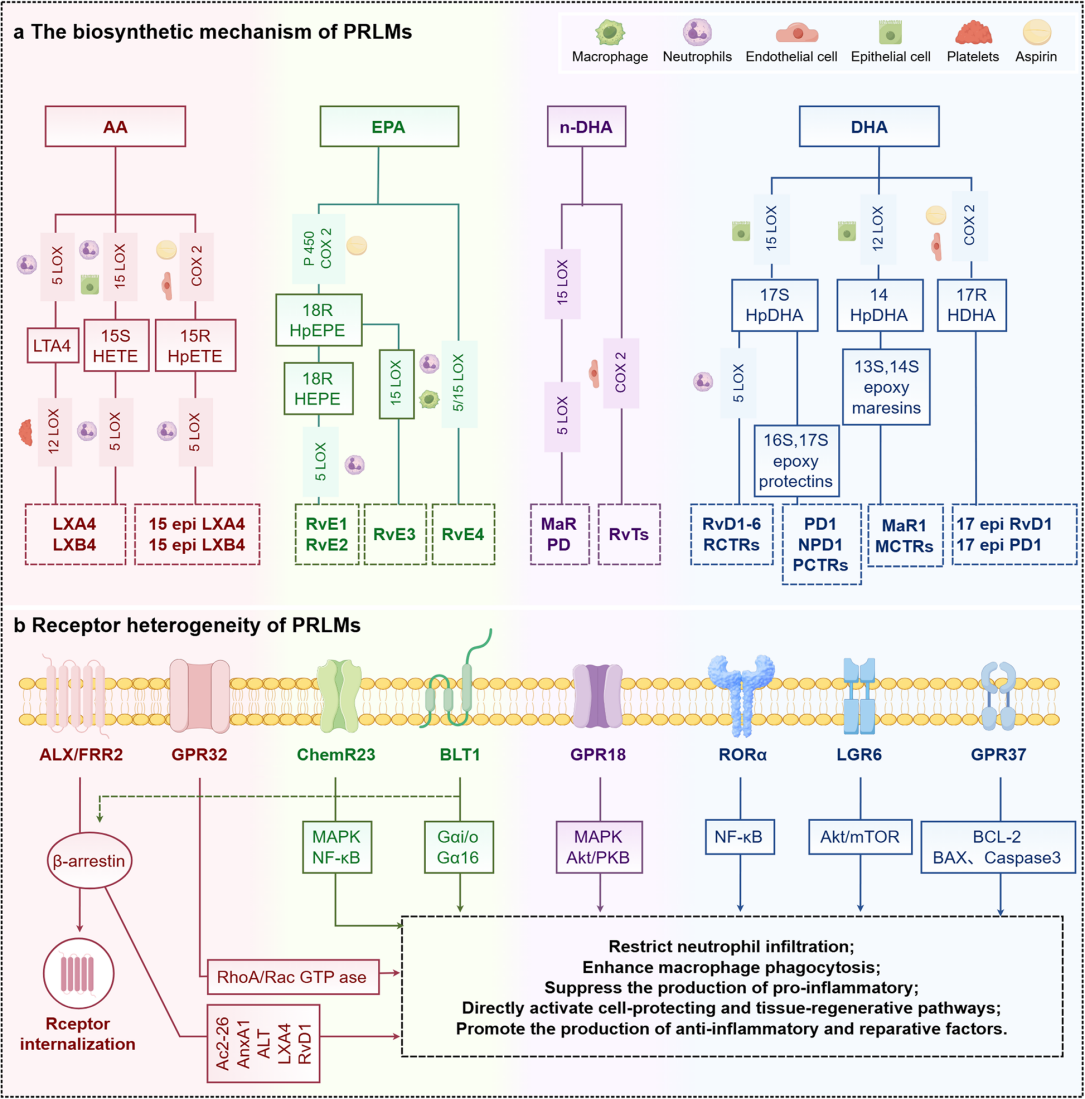
Biosynthetic pathways and receptor heterogeneity of PRLMs (By Figdraw, License ID: UYWWS424ef). **a** Inflammatory phase: Tissue injury activates vascular endothelium and promotes neutrophil infiltration through Mac-1–integrin–mediated transendothelial migration. Activated neutrophils release reactive oxygen species (ROS), myeloperoxidase, and DAMPs, leading to vascular leakage and amplification of inflammation via NADPH oxidase, PKC, and MAPK signaling. **b** Resolution phase: Pro-resolving lipid mediators (PRLMs) inhibit leukocyte adhesion and NF-κB–driven inflammation, promote efferocytosis and M2 macrophage polarization through cAMP/PKA and AKT/mTOR pathways, and induce tolerogenic dendritic cells and Foxp3⁺ Treg activation. These processes enhance IL-10 and TGF-β release and restore immune homeostasis. Abbreviations: PRLMs, pro-resolving lipid mediators; ROS, reactive oxygen species; DAMPs, damage-associated molecular patterns; PKC, protein kinase C; MAPK, mitogen-activated protein kinase; NF-κB, nuclear factor kappa-B; PKA, protein kinase A; DCs, dendritic cells; Treg, regulatory T cell; ICAM, intercellular adhesion molecule

PRLMs originate from PUFAs and are categorized into four major families: LXs, Rvs, PDs, and maresins (MaRs). These mediators are endogenously generated by immune and vascular endothelial cells (Fig. 4). Their production depends on specific PUFA substrates, including arachidonic acid (AA), eicosapentaenoic acid (EPA), n-3 docosapentaenoic acid (n-3 DPA), and docosahexaenoic acid (DHA). Biosynthesis involves a coordinated sequence of enzymatic reactions primarily regulated by 5-lipoxygenase (5-LOX), 12-LOX, 15-LOX, cyclooxygenase-2 (COX-2), cytochrome P450 enzymes (CYP), and epoxide hydrolase [12, 13].

Among these families, LXs were the first lipid-derived PRLMs identified and are primarily synthesized from AA [14]. Their biosynthesis requires multiple lipoxygenase (LOX) enzymes. In epithelial cells and monocytes, AA is metabolized by 15-LOX, whereas neutrophils generate LX through 5-LOX activity. Platelets further contribute to LX formation through interactions with leukocytes, where 12-LOX converts leukotriene A4 into LX. In addition, aspirin-acetylated COX-2 can cooperate with 5-LOX to produce LX, offering mechanistic insight into the pro-resolving effects of aspirin [15].

Rvs, another major family, are ω−3 PUFA–derived PRLMs generated from EPA and DHA and are classified into E-series (RvEs) and D-series (RvDs) [12]. RvE biosynthesis predominantly depends on 5-LOX and involves several pathways. RvE1 and RvE2 are produced from EPA via either an aspirin-dependent route requiring acetylated COX-2 in cooperation with 5-LOX in neutrophils [16], or an aspirin-independent pathway involving CYP enzymes and 5-LOX [17]. RvE3 is formed from the intermediate 18R-hydroxyeicosapentaenoic acid via a 15-LOX–dependent mechanism [18]. Under hypoxic conditions, RvE4, a recently identified member, is synthesized in neutrophils and macrophages via either 15-LOX or 5-LOX [18]. The biosynthesis of RvDs requires sequential enzymatic processing by 15-LOX and 5-LOX, which convert DHA into 17S-hydroxydocosahexaenoic acid and subsequently generate Resolvin D1 (RvD1) through RvD6 [19, 20]. Aspirin-triggered RvDs (AT-RvDs) are similarly derived from DHA in the presence of acetylated COX-2 [12]. Recently, three subclasses of cysteinyl-conjugated PRLMs, collectively termed cysteinyl-PRLMs (cys-PRLMs), have been identified [21]. Within this group, tissue-regenerative Rv conjugates are generated from DHA via 15-LOX activity [21].

PDs are synthesized from DHA via a 15-LOX–dependent pathway. During this process, the intermediate 16S,17S-epoxy-PD is hydrolyzed to generate PD1 and related PD conjugates, which contribute to tissue regeneration by regulating neutrophils, macrophages, eosinophils, and T cells [21]. When produced by neurons or retinal pigment epithelial cells, PD1 is called neuroprotectin D1 (NPD1) [22]. MaRs, originally described as “macrophage mediators in resolving inflammation,” represent another DHA-derived PRLM family [12]. Their biosynthesis begins with the 12-LOX–mediated conversion of DHA to 14-hydroperoxy-DHA, followed by the formation of 13S,14S-epoxy intermediates and ultimately MaR1 and MaR2. The 13S,14S-epoxy intermediate may undergo additional modification by glutathione S-transferase μ4 and leukotriene C4 synthase to yield maresin conjugates involved in tissue regeneration (MCTRs) [21]. More recently, n-3 DPA has been identified as a precursor of four additional PRLMs subclasses: Rv n-3 DPA, PD n-3 DPA, MaR n-3 DPA, and 13-series resolvins (RvTs) [23]. Defining the enzymatic pathways underlying the biosynthesis of each subtype is essential for advancing mechanistic insights into dysregulated inflammation and guiding future therapeutic development.

ω−3 fatty acids cannot be synthesized by mammals and must be obtained through diet. Recent studies using Fat-1 transgenic mice, which possess the ability to convert ω−6 fatty acids into ω−3 fatty acids, have provided new mechanistic insights [24]. Under high-fat diet conditions, Fat-1 mice exhibit marked metabolic protection, including a reduced risk of non-alcoholic fatty liver disease (NAFLD), attenuated inflammation in brown adipose tissue (BAT), and improved glucose tolerance [25]. These findings highlight the importance of substrate availability for PRLM biosynthesis and indicate that increasing PRLM levels through dietary interventions or metabolic regulation may represent a promising therapeutic strategy for inflammation-related diseases.

### PRLM receptors and their intracellular signaling circuits

PRLMs exert pro-resolving effects primarily by binding to specific GPCRs within the transmembrane receptor superfamily [26]. GPCR-based screening has identified multiple receptors that respond to PRLMs (Fig. 4) [26]. This ligand–receptor interactions are not one-to-one; a single receptor may recognize multiple PRLMs, and conversely, one PRLMs can activate more than one receptor. For example, RvD1 [27], RvD3 [28], and RvD5 [29] all interact with GPR32, whereas RvD1 can also bind to the Annexin A1 receptor/formyl peptide receptor 2 (ALX/FPR2), although it displays a higher affinity for GPR32 [15]. Because mice lack the GPR32 gene, RvD1 signaling in murine studies is assumed to occur through ALX/FPR2 [15].

Despite engaging distinct receptors, PRLMs usually induce similar functional outcomes, including enhanced inflammatory resolution and tissue repair. This convergence suggests that the downstream signaling pathways may overlap or exhibit mechanistic crosstalk. Some PRLM receptors can also bind to proinflammatory mediators, demonstrating ligand-dependent functional duality. For instance, the recently identified receptor for MCTRs, the cysteinyl leukotriene (cysLT) receptor, also binds leukotrienes D4 and E4 (LTD4 and LTE4) [30, 31]. Similarly, ALX/FPR2, which recognizes both lipoxin A4 (LXA4) and RvD1, can be activated by serum amyloid A, triggering the phosphoinositide-3 kinase (PI3K) and nuclear factor-κB (NF-κB) signaling pathways and promoting inflammation [32]. These findings highlight that receptor signaling outcomes depend on the ligand context, whether pro-resolving or proinflammatory, and may reflect distinct binding domains or biased signaling programs.

Besides GPCRs, PRLMs engage additional receptor classes. For example, LXA4 activates the aryl hydrocarbon receptor [32] and cannabinoid receptor 1 [33], exerting anti-inflammatory and neuroprotective effects. MaR1 also activates the orphan nuclear receptor RORα, promoting an anti-inflammatory macrophage phenotype [34]. In neural and sensory systems, PRLMs regulate nociception via transient receptor potential (TRP) ion channels. Specifically, they suppress TRPV1 [35], reducing acute pain response.

### Molecular networks underlying the cellular functions of PRLMs

Functional studies have further clarified that PRLMs act mainly through receptor-dependent mechanisms, particularly by activating GPCRs, to exert broad immunomodulatory effects [36]. For example, PRLMs suppress dendritic cell (DC) maturation and migration, reduce proinflammatory cytokine release, and prevent excessive T-cell activation [36]. Simultaneously, they promote regulatory T-cell (Treg) differentiation and increase the production of TGF-β and IL-10, attenuating persistent inflammation. In macrophages, PRLMs regulate morphology and function via receptor-mediated signaling, enhancing phagocytic capacity and driving polarization toward a pro-repair phenotype [37]. At the B cell level, supplementation with PRLMs or their precursor PUFAs enhances antibody production, strengthening the humoral immune barrier that restricts inflammatory progression [38]. Collectively, these findings illustrate the synergistic effects of PRLMs across diverse immune cell populations and emphasize their central role in maintaining immune homeostasis [39]. Evidence from animal studies shows that MaR1, RvD1, RvD2, and RvE1 inhibit excessive proliferation and migration of vascular smooth muscle cells (VSMCs), downregulate adhesion molecules such as vascular cell adhesion molecule-1 (VCAM-1) and intercellular adhesion molecule-1 (ICAM-1), and reduce both reactive oxygen species (ROS) production and proinflammatory mediator release [40, 41]. These actions are associated with the suppression of the NF-κB signaling pathway and activation of the cyclic adenosine monophosphate (cAMP)-protein kinase A (PKA) pathway, highlighting the therapeutic potential of PRLMs in vascular remodeling and inflammation-related diseases [42].

PRLMs regulate immune cell function, remodel cytokine networks, and promote tissue repair through multi-target synergistic actions, serving as central mediators of immune homeostasis [43]. Advances in lipidomics, AI-driven receptor prediction, and structural biology are expected to accelerate the discovery of novel PRLMs and their receptors, while also providing deeper insights into their metabolic regulation [44]. Collectively, these developments will broaden our understanding of inflammatory homeostasis and establish a solid theoretical basis for designing resolution-based precision interventions and promoting their clinical translation.

## Advances in PRLMs across diseases and their clinical implications

**Fig. 5:**
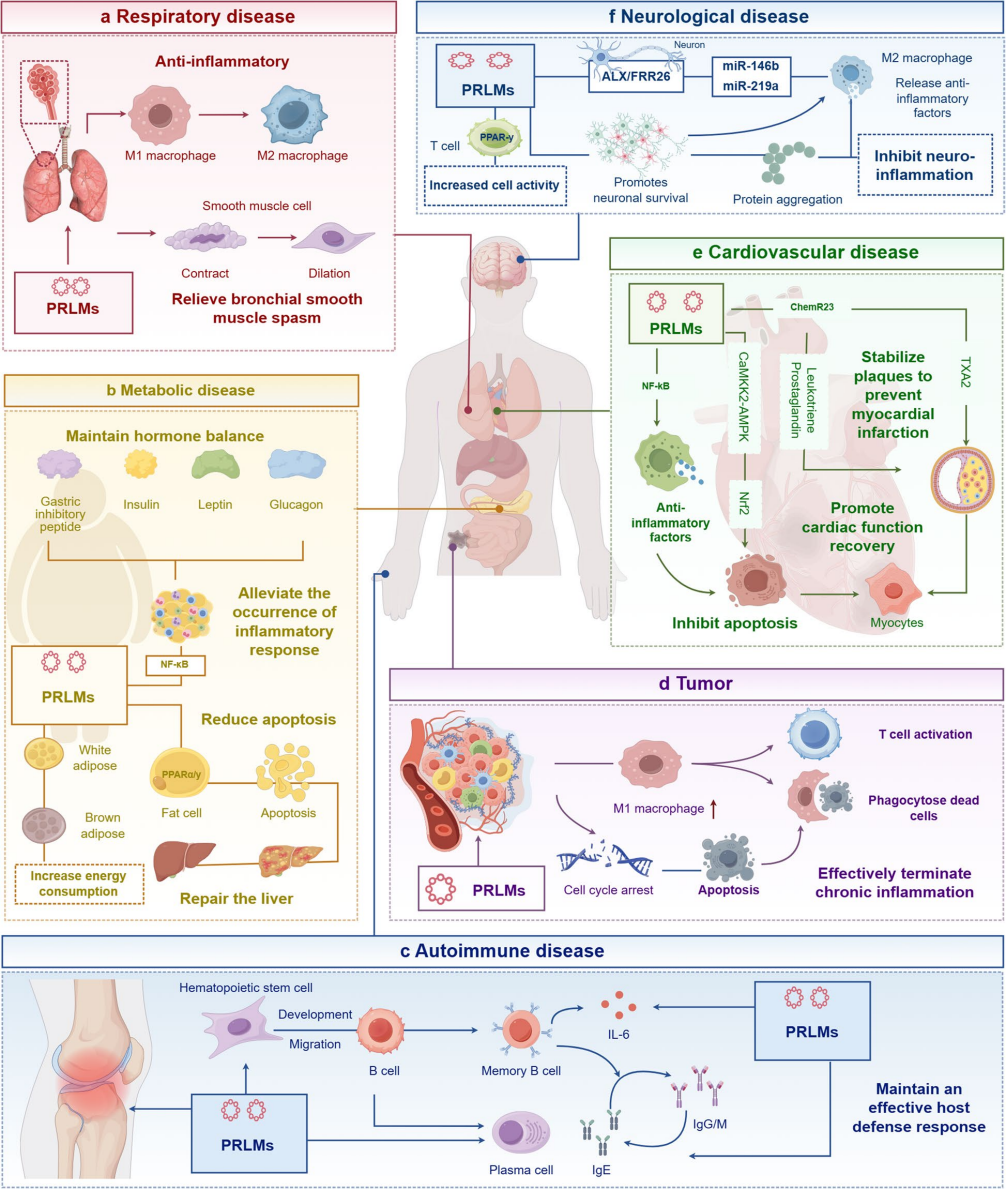
Biological functions and therapeutic implications of PRLMs across major disease systems (By Figdraw, License ID:WSRPR86373). **a** Respiratory diseases: PRLMs regulate airway inflammation by promoting M2 macrophage polarization, suppressing M1-driven responses, and relieving bronchial smooth muscle spasm. **b** Metabolic diseases: PRLMs modulate hormonal homeostasis, restrain NF-κB–mediated inflammation, reduce adipocyte apoptosis, enhance lipid metabolism and energy expenditure, and contribute to hepatic repair. **c** Autoimmune disease: PRLMs modulate B cell hyperactivation and promote antibody production, such as immunoglobulin (Ig) G and M, to maintain an effective host defense response; **d** Tumors: PRLMs mitigate chronic inflammation and tumor progression by enhancing efferocytosis, activating T cells, and inducing apoptosis and cell cycle arrest in malignant cells. **e** Cardiovascular diseases: PRLMs stabilize atherosclerotic plaques, promote cardiac protection and functional recovery, and inhibit apoptosis through ChemR23– and CaMK/AMPK–dependent signaling pathways. **f** Neurological diseases: PRLMs improve neuronal survival, reduce protein aggregation, and attenuate neuroinflammation via ALX/FPR2– and PPARγ-regulated signaling and upregulation of miR-146b and miR-219a. Abbreviations: PRLMs, pro-resolving lipid mediators; NF-κB, nuclear factor kappa-B; AMPK, AMP-activated protein kinase; CaMK, Ca^2^⁺/calmodulin-dependent protein kinase; PPARγ, peroxisome proliferator-activated receptor γ; ALX/FPR2, Annexin A1 receptor/Formyl peptide receptor 2; TXA₂, thromboxane A₂; M1/M2, macrophage phenotypes

In diverse disease contexts, PRLMs are essential for maintaining immune homeostasis by precisely regulating the initiation and resolution phases of inflammation. Once early proinflammatory responses achieve pathogen or debris clearance, PRLMs shift the immune system toward resolution within a defined timeframe. During this phase, they promote the removal of apoptotic cells and necrotic tissue and coordinate interactions among immune, vascular, and parenchymal cells, fostering tissue repair and functional recovery [45]. Progress in lipidomics and dynamic monitoring technologies has further elucidated PRLM-related pathway activity and clarified their clinical significance, providing new opportunities for patient stratification, disease evaluation, and therapeutic optimization [46]. Based on this, the following sections examine chronic inflammatory diseases, systematically evaluating the roles and clinical implications of PRLMs in pathological conditions (Fig. 5). Table 1 summarizes the recent applications of PRLMs in systemic diseases.

**Table 1:** Applications of PRLMs in systemic disease

Disease	PRLMs studied	Effects	Sample/Species	Administration	Refs
RA	RvD1	Promotes cartilage/bone damage	Human	Oral administration	[47]
	MCTR3	Anti-inflammatory and joint-repair effects	Mice	Tail vein injection	[48]
IBD	RvD2	Inhibits neutrophil infiltration	Mice	Oral administration	[49]
	PD1, RvD5	Reduce colonic damage	Mice	Intraperitoneal injection	[50]
Myocarditis	BML-111	Alleviate myocarditis and prevent cardiac dysfunction	Mice	Intraperitoneal injection	[51]
	LXA4	Prevents cardiac apoptosis	Mice	Intramyocardial administration	[52]
	RvE1	Reduces IL-1β secretion	Mice	Oral administration	[53]
	AT-RvD1	Clears adverse inflammatory environments	Mice	Oral administration	[54]
	LXA4	Suppresses myocardial fibroblast inflammation	CFs	Co-culture	[55]
	RvE1, RvD1	Inhibit ICAM-1 and VCAM-1 proteins	CFs	Co-culture	[56]
AD	LXA4	LXA4 deficiency correlates strongly with cognitive decline	/	Human	[57]
	MaR1	Enhanced interest in neuronal survival	Micro-glia	Co-culture	[58]
Parkinson’s disease	RvD1	Recovery of the DA neurotransmission	Rat	Intraperitoneal injection	[59]
MS	LXA4	Reduced brain-derived Th 1 and Th 17 effector functions	Mice	Intraperitoneal injection	[51]
Obesity	MaR1	Improved insulin sensitivity	Mice	Subcutaneous injection	[60]
	LXA4	Preventing diet-induced obesity and insulin resistance in mice	Mice	/	[61]
	RvD1, RvD2	Counteract local adipokine production and monocyte accumulation	Mice	/	[62]
	DHA	Reduce the BAT functional impairment	Mice	Oral administration	[63]
	RvD2	Promote the expression of UCP 1 and PGC1α in the BAT	Mice	Intracerebroventricular administration	[64]
NAFLD	PD1, 17-HDHA	Mains inflammation in adipose tissue and promotes insulin sensitivity	Mice	Osmotic pump	[65]
Prostatic cancer	LXA4	Activation of the STAT6 pathway	Mice	Subcutaneous injection	[66]
Osteocarcinoma	LXs	Strong analgesic effect	Rat	Intrathecal or intravenous injection	[67]
Pancreatic cancer	LXA4	By inhibition of pSmad2/3 signaling	hPSCs	Co-culture	[68]
Lung cancer	LXA4	Inhibition of the immune cell recruitment	Mice	Intravenous injection	[69]
AS	15-epi-LXA4	Blocked atherosclerosis progression	Mice	Injected with AAV8-gof-PCSK9	[54]
	RvD1	Reduce plaque necrosis	Mice	Exogenous administration of RvD1 to macrophages	[70]
	RvD1	Attenuates atherosclerosis	Mice	Intraperitoneal injection	[71]
	RvD2, MaR1	Promotes plaque stability	Mice	Intraperitoneal injection	[72]
	RvE1	Markedly reduced intimal hyperplasia	Mice	Intraperitoneal injection	[40]
	RvD1	Reduced neointimal formation	Rat	Pluronic F127 gels	[41]
	RvD2, MaR1	Decreased intimal hyperplasia	Mice	Intraperitoneal injection	[73]
Peripheral artery Disease	LXA4	Markedly ameliorated histological damage	Rat	Tail vein injection	[74]
	RvD2	Stimulates arteriogenic revascularization	Mice	Ligation site subcutaneous injection	[75]
	RvD1, PD1	Reduced neointimal formation	Mice	Tail vein injection	[76]
Hypertension	RvD2	Prevents hypertension and vascular dysfunction	Mice	Intraperitoneal injection	[77]
MI	15-epi-LXA4	Reducing LV dysfunction	Mice	Liposomal delivery	[78]
	RvD1	Limiting cardiac remodeling	Mice	Liposomal delivery	[79]
	RvE1	Ameliorating cardiac function	Mice	Tail vein injection	[80]
	MaR1	Improved cardiac function	Mice	Intraperitoneal injection	[81]
	RvE1	Reduces infarct size	Rat	Tail vein injection	[82]
	12/15- LOX	Limiting cardiac remodeling	Mice	Oral administration	[83]
Asthma	RvD1	Reduce eosinophil infiltration	Mice	Intravenous injection	[84]
	RvE1	Alleviate airway hyperreactivity	Mice	Intraperitoneal injection	[85]
	PUFAs	Reduce the incidence of asthma	Human	Oral administration	[86]
	LCPUFA	The risk of persistent wheezing in offspring asthma was markedly reduced by 30.7%	Human	Oral administration	[87]
COPD	PUFA	PUFA supplementation markedly improved exercise capacity	Human	Oral administration	[88]
	EPA, DPA	Supplementation of EPA (p = 0.006) and DPA (p = 0.022) was significantly associated with a slower FEV1 decline	Human	Oral administration	[89]
CF	LTB4	One study reported reduced pulmonary exacerbations and antibiotic use when the subjects were treated with PUFAs. In another 6-week study, sputum levels were reduced, and lung function and clinical status improved when taking ω−3 supplements	Human	Oral administration	[90]
COVID-19	PUFAs	Patients with COVID-19 who were critically ill showed improved respiratory and renal function, leading to higher 1-month survival rates	Human	Oral administration	[91]
	Fatty acids	ω−3 supplements reduced the risk of COVID-19 by 12%–21%. However, a deficiency in ω−3 has been linked to severe COVID-19 symptoms, a higher need for mechanical ventilation, hospitalization, and increased mortality	Human	Oral administration	[92]
SLE	RvD1	Reduced disease severity, decreased total IgG, anti-dsDNA, and ANA levels in the serum	Mice	Intravenous injection	[93]
	RvD1	Decreased RvD1 in the plasma from patients with SLE compared with those of healthy controls	Human	-	[94]
Acute inflammatory	RvD1	Reduced disease severity, body weight loss, and demyelination	Rat	Intraperitoneal injection	[95]
Nephritis	15-epi-LXA4	Decreased neutrophils infiltration in the glomeruli	Mice	-	[96]
HT	RvD1	Decreased RvD1 and RvE1 levels in the serum from patients with HT compared with those of healthy controls	Human	-	[97]
NMOSD	RvD1	Decreased RvD1 levels in the CSF from patients with NMOSD compared with those of healthy controls	Human	-	[98]

*Abbreviations*: *AD* Alzheimer’s disease, *ALX/FPR2* Annexin A1 receptor/Formyl peptide receptor 2, *AMPK* AMP-activated protein kinase, *ANA* ntinuclear antibody, *AS* Atherosclerosis, *BAT* Brown adipose tissue, *CaMKK2* Calcium/calmodulin-dependent protein kinase 2, *ChemR23* Chemerin receptor 23, *CF* Cystic fibrosis, *CFs* Cardiac fibroblasts, *COPD* Chronic obstructive pulmonary disease, *CSF* Cerebrospinal fluid, *DPA* Docosapentaenoic acid, *EPA* Eicosapentaenoic acid, *ERK1/2* Extracellular signal-regulated kinase 1/2, *FEV1* Forced expiratory volume in 1 s, *GPR40* G-protein–coupled receptor 40, *GPR120* G-protein–coupled receptor 120, *HT* Hashimoto’s thyroiditis, *HO-1* Heme oxygenase 1, *IBD* Inflammatory bowel disease, *ILD* Inflammatory lung disease, *IRAK1* Interleukin-1 receptor-associated kinase 1, *LCPUFAs* Long-chain polyunsaturated fatty acids, *LXA4* Lipoxin A4, *MCTR3* Maresin conjugate in tissue regeneration 3, *MS* multiple sclerosis, *NAFLD* Non-alcoholic fatty liver disease, *NASH* Non-alcoholic steatohepatitis, *NF-Κb* Nuclear factor kappa-light-chain-enhancer of activated B cells, *NMOSD* Neuromyelitis optica spectrum disorders, *NRF2* Nuclear factor erythroid 2-related factor 2, *PD1* Protectin D1, *PI3K* Phosphoinositide 3-kinase, *PKB* Protein kinase B, *PRLMs* Pro-resolving lipid mediators, *PUFAs* Polyunsaturated fatty acids, *RA* Rheumatoid arthritis, *RvD1* Resolvin D1, *RvD2* Resolvin D2, *RvE1* Resolvin E1, *RORα* Retinoic acid receptor–related orphan receptor alpha, *SLD* Scleroderma lung disease, *SLE* Systemic lupus erythematosus, *TGF-β* Transforming growth factor beta, *THRβ* Thyroid hormone receptor β, *ω−3* Omega-3, *5-LOX* Lacking 5-lipoxygenase

### Cardiovascular disorders: PRLMs in inflammation resolution and tissue repair

Following injury caused by ischemia, hypoxia, inflammatory mediators, physical trauma, chemical insults, biological infection, or drug-induced cardiotoxicity, the heart rapidly initiates an immune-inflammatory response to clear necrotic tissue and maintain homeostasis [4]. This response unfolds in two phases: a proinflammatory phase and a resolution/repair phase. During the proinflammatory phase, necrotic cells release damage-associated molecular patterns, proteases, lysosomal enzymes, and mitochondrial ROS, which activate local immune responses and recruit monocytes and neutrophils from the spleen and circulation to the injured myocardium. In parallel, resolution pathways are progressively activated, with enhanced synthesis of endogenous PRLMs that actively promote inflammation resolution and initiate tissue repair. During the repair phase, infiltrating monocytes and neutrophils clear apoptotic cells and cellular debris through phagocytosis and secrete repair factors to restore tissue integrity. When this resolution process is impaired, sustained and excessive inflammation drives maladaptive myocardial remodeling and may ultimately result in heart failure (HF). Thus, clarifying how PRLMs orchestrate inflammation resolution and myocardial repair is crucial for advancing PRLM-based precision therapies [13].

#### Ischemic heart disease (IHD)

IHD is the most prevalent cardiovascular disorder, defined by a pathological cascade involving ischemia, reperfusion, and inflammation-induced injury [99]. Hypoxia and nutrient deprivation damage myocardial cells and activate the inflammatory response. Although reperfusion, whether spontaneous or achieved through percutaneous coronary intervention, restores blood flow, it simultaneously provokes ischemia–reperfusion injury (IRI) through a sudden burst of ROS, exacerbating myocardial damage. Early inflammation is indispensable for initiating tissue repair; however, if sterile inflammation persists without timely resolution, it drives myocardial fibrosis and maladaptive remodeling, ultimately leading to poor outcomes. Thus, the balance between proinflammatory activity and PRLM-mediated resolution is a critical determinant of the myocardial injury prognosis [100].

Clinical studies further support the relevance of PRLMs in IHD, highlighting their diagnostic and prognostic value. In patients with acute coronary syndrome (ACS), plasma RvD1 levels are markedly reduced, whereas leukotriene B4 (LTB4) levels are elevated; notably, the RvD1/LTB4 ratio is closely associated with ACS risk [101]. In acute myocardial infarction (AMI), higher levels of LXA4 are inversely correlated with the incidence of major adverse cardiac events (MACE) [102]. Dynamic monitoring using liquid chromatography–tandem mass spectrometry (LC–MS/MS) has shown a transient rise in plasma PRLMs in patients with ST-elevation myocardial infarction (STEMI) before the peak of cardiac troponin T (cTnT), followed by a rapid decline [103]. This pattern suggests that acute inflammation may activate the compensatory synthesis of PLRMs. Moreover, low RvD1 levels at admission in patients with AMI are associated with adverse clinical outcomes [104]. Collectively, these findings underscore the central role of PRLM-mediated resolution in post-ischemic myocardial repair and support its potential application as a biomarker for clinical risk assessment.

Complementary evidence from animal studies has provided mechanistic insights into how PRLMs confers cardioprotection. Both local and systemic administration of RvD1 markedly reduced myocardial infarct size [105]. These benefits are mediated through the downregulation of the high mobility group box 1 (HMGB1)/toll-like receptor 4 (TLR4)/NF-κB inflammatory pathway, activation of PI3K/protein kinase B (Akt) pro-survival signaling, and inhibition of excessive neutrophil infiltration via ALX/FPR2 receptors in the spleen and left ventricle, limiting uncontrolled inflammation [106]. In addition, RvD1 promotes the synthesis of other PRLMs, including RvD2, MaR1, and LXA4, amplifying pro-resolving signaling. Similarly, intravenous administration of RvE1 before ischemia markedly decreased infarct size and prevented apoptosis through the phosphorylation of Akt and extracellular signal-regulated kinase 1/2 (ERK1/2) [107]. Subsequent studies have identified a therapeutic window for RvE1 after AMI: treatment within 1–7 days improves cardiac function, whereas administration between 7 and 14 days may suppress angiogenesis and delay recovery [82].

Besides Rvs, other PRLMs also demonstrate potent cardioprotective effects. Liposomal 15-epi-LXA4 accelerates the resolution of inflammation by activating FPR2/GPR120 and inhibiting G-protein–coupled receptor 40 (GPR40), alleviating left ventricular dysfunction. MaR1 improves ventricular remodeling and reduces arrhythmias by activating the nuclear factor erythroid 2-related factor 2 (NRF2)/heme oxygenase-1 (HO-1) pathway and suppressing TLR4/NF-κB signaling, while also mitigating myocardial hypertrophy via the retinoic acid–related orphan receptor alpha (RORα)/insulin-like growth factor 1 (IGF-1)/PI3K/Akt axis [108]. Together, these findings indicate that PRLMs function not only as passive anti-inflammatory agents but also as active drivers of repair by reprogramming immune cell function, enhancing necrotic tissue clearance, and promoting regeneration [80]. Studies of IHD highlight the dual role of PRLMs in inflammation resolution, tissue repair, and clinical translation. Dynamic profiling of PRLMs may serve as a novel tool for early diagnosis and risk stratification in AMI and ACS. However, exogenous supplementation or the development of stable analogs offers a promising avenue to overcome the limitations of conventional anti-inflammatory strategies and deliver more precise therapies for ischemic cardiac injuries.

#### Intimal hyperplasia (IH)

IH is the most common and challenging form of pathological remodeling after vascular injury and serves as the principal pathological basis for restenosis following balloon angioplasty, stent implantation, arteriovenous fistula creation, and bypass grafting [109]. It begins with mechanical damage to the vascular endothelium, triggering platelet aggregation and activation. Activated platelets release chemotactic factors, such as platelet-derived growth factor (PDGF), epidermal growth factor (EGF), and TGF-β, which recruit monocytes, neutrophils, and other immune cells to the site of injury. These infiltrating cells secrete large amounts of proinflammatory cytokines [109]. In this sustained inflammatory environment, VSMCs switch from a differentiated contractile phenotype to a synthetic phenotype, marked by enhanced proliferation, migration toward the vascular lumen, and production of ECM. This process ultimately causes intimal thickening and luminal narrowing [110]. If inflammation fails to resolve, delayed endothelial repair and persistent immune cell infiltration perpetuate a vicious cycle of inflammatory imbalance and vascular remodeling, driving the development of restenosis.

Although drug-eluting stents and balloon angioplasty are widely used to suppress IH, their therapeutic efficacy remains limited, with persistent challenges such as incomplete inflammation control and impaired local repair [111]. The discovery of PRLMs has introduced a promising strategy for overcoming these limitations. Beyond their role in the resolution phase of inflammation, PRLMs exert multidimensional effects, including immunoregulation, endothelial repair, and VSMC phenotypic remodeling. Evidence from lower limb ischemic injury models shows that local levels of RvD2 and its biosynthetic precursor 17-H(p)DHA increase markedly after vascular injury, suggesting that the body spontaneously synthesizes PRLMs to promote inflammation resolution and tissue regeneration [75]. Exogenous administration of RvD2 further enhances these protective effects by suppressing inflammation and accelerating repair, highlighting its critical role in maintaining local homeostasis after injury [112].

In multiple animal models, PRLMs have demonstrated broad and consistent protective effects. In balloon-injured rabbit arteries, the RvD series markedly inhibited vascular remodeling by suppressing VSMC proliferation and leukocyte recruitment [113]. These protective effects were linked to the local upregulation of RvD1 receptors FPR2 and GPR32, along with positive feedback synthesis of endogenous PRLMs [113]. In a carotid artery ligation mouse model, RvD2 and MaR1 markedly reduced VSMC proliferation, limited neutrophil and macrophage infiltration, and exerted antifibrotic effects by promoting macrophage polarization toward the reparative M2 phenotype [73]. Similarly, AT-LXA4 inhibited VSMC migration by binding to the FPR2/ALX receptor; however, this effect was abolished in receptor-deficient mice [114]. RvE1 also acts via the chemokine-like receptor 1 (ChemR23) to block VSMC migration, restrict neutrophil and T-cell recruitment, and enhance M2 polarization, markedly alleviating IH in a femoral artery injury mouse model [40]. Taken together, these findings underscore the central role of PRLMs in regulating vascular inflammation and support their potential as precise therapeutic strategies for preventing restenosis.

The route of PRLM administration has a decisive impact on therapeutic efficacy. Localized delivery offers greater efficacy and safety than systemic administration. In a rat carotid angioplasty model, local delivery of RvD1 using a biodegradable Pluronic F127 hydrogel markedly suppressed NF-κB activation, reduced VSMC proliferation and migration, and effectively attenuated IH [41]. In a mouse aortic bypass model, incorporation of the ALX peptide mimetic AT-RvD1 into polycaprolactone (PCL) vascular grafts markedly inhibited neutrophil infiltration into the graft wall and promoted macrophage polarization toward the anti-inflammatory M2 phenotype, accelerating re-endothelialization and tissue repair [115]. By contrast, oral administration of RvD1 in a rat carotid angioplasty model produced modest anti-inflammatory effects but failed to markedly inhibit IH, underscoring the critical importance of developing targeted delivery strategies to achieve successful clinical translation [116].

With ongoing research, the role of PRLMs has expanded from a single anti-inflammatory action to multidimensional interventions, including immune remodeling, VSMC phenotype regulation, and endothelial regeneration. When combined with emerging strategies, such as localized delivery systems, biodegradable stents, and targeted nanoscale platforms, PRLMs demonstrate strong potential for clinical translation [4, 10]. These developments deepen our understanding of vascular repair mechanisms and open new opportunities to improve long-term outcomes after cardiovascular interventions. Future studies should focus on clarifying the specific roles of different PRLM subtypes, integrating them with precision delivery strategies, and advancing early phase clinical trials to translate basic discoveries into effective therapies for vascular restenosis.

#### Hypertension

Hypertension results from hemodynamic abnormalities and is increasingly recognized as a systemic disorder driven by chronic low-grade inflammation [117]. Substantial evidence demonstrates that patients with hypertension exhibit pronounced systemic inflammatory responses characterized by elevated levels of proinflammatory mediators, such as IL-6, TNF-α, and C-reactive protein (CRP), along with a marked imbalance in anti-inflammatory pathways and PRLMs [118]. This persistent inflammatory dysregulation accelerates vascular remodeling and cardiovascular dysfunction, establishing a pathological basis for hypertension-related complications.

Because patients with hypertension exhibit persistent activation of inflammatory signaling and impaired resolution capacity, conventional antihypertensive drugs alone are insufficient to restore vascular function. In this context, PRLMs have emerged as a promising therapeutic strategy due to their ability to promote inflammation resolution, protect the vascular endothelium, and reverse vascular remodeling. Evidence increasingly indicates that specific PRLMs play pivotal roles in the onset and progression of hypertension by improving vascular function, modulating immune responses, and suppressing vascular remodeling. Among them, RvE1 exerts multifaceted anti-inflammatory and immunomodulatory effects by binding to ChemR23 (also referred to as LTB4 receptor BLT1). It enhances macrophage phagocytosis, disrupts thromboxane A₂ (TXA₂)-mediated platelet aggregation, and inhibits neutrophil migration to the vascular walls. At the same time, it regulates macrophage chemotaxis and cytokine production, facilitating resolution of the inflammatory microenvironment [119]. In addition, RvE1 protects vascular endothelial cells by limiting leukocyte adhesion and transmigration, mitigating systemic inflammation–induced endothelial dysfunction, and preserving vascular barrier integrity by regulating microvascular permeability [120].

Building on these findings, recent animal studies have confirmed that RvD1, RvE1, and RvD2 provide markedly cardiovascular protection in hypertensive models. RvD1 and RvE1 induce arterial smooth muscle relaxation in both rats and humans [77]. RvD2 treatment partially prevents angiotensin II (Ang II)-induced hypertension, lowers blood pressure and vascular stiffness, reduces myocardial fibrosis, and decreases neutrophil infiltration [77]. In spontaneously hypertensive rats (SHRs), Camilla F and colleagues observed a marked reduction in endogenous PRLM precursors, resulting in impaired biosynthesis and delayed resolution of inflammation, a phenomenon closely associated with increased lipid peroxidation in resistance arteries [121]. Under these conditions, exogenous PRLM supplementation markedly improved endothelial function, primarily through the activation of the FPR2 receptor. This intervention suppressed TNF-α–induced ROS generation and restored acetylcholine-dependent vasodilation in mouse arteries [121].

The role of PRLMs has also been explored in metabolic inflammation–related hypertension. Ana et al. reported that in mice with hypertension induced by a high-fat diet combined with Ang II, RvD2 partially prevented blood pressure elevation and reduced vascular proinflammatory markers and apoptosis [122]. In human macrophages, RvD2 reversed Ang II–induced impairment of phagocytic function and reshaped the PRLM profile, underscoring its bidirectional regulatory potential in hypertension-associated immune imbalance [122]. Additional animal studies have shown that exogenous PRLM supplementation lowers blood pressure and markedly reduces vascular wall inflammation and endothelial injury, improving cardiovascular structure and function [122]. Despite this robust preclinical evidence, studies on human hypertension remain at an early exploratory stage, highlighting the need for large-scale randomized controlled trials to establish clinical efficacy.

Although PRLMs show considerable therapeutic potential for hypertension, their clinical translation remains constrained by several challenges [123]. First, their short half-life and rapid metabolism limit the ability to maintain stable and effective in vivo concentrations. To overcome this, future research should focus on developing advanced delivery systems, such as lipid nanoparticles (LNPs), exosome carriers, or receptor-targeted strategies, to improve bioavailability and tissue specificity. Second, the actions of different PRLM subtypes do not entirely overlap, as they differ in receptor affinities (e.g., FPR2 and ChemR23) and downstream signaling pathways. Clarifying these subtype-specific mechanisms is critical for precision therapy. Finally, integrating multi-omics approaches, including lipidomics, single-cell transcriptomics, and spatial omics, with dynamic monitoring may provide insights into the regulation of hypertension-associated inflammatory networks and aid in identifying biomarkers for patient stratification and therapeutic response prediction [123].

PRLMs not only represent promising molecular targets for inflammation resolution–based therapies in hypertension but also hold potential for advancing the precision management of patients with hypertension. With continued advances in delivery technologies and receptor-specific drug development, PRLMs are poised to move toward clinical practice and may transform therapeutic strategies for cardiovascular diseases.

#### Atherosclerosis (AS)

AS is widely recognized as a chronic immune-inflammatory disease initiated and driven by complex interactions among vascular endothelial cells, smooth muscle cells, macrophages, and circulating monocytes [116]. In the early stages, low-density lipoprotein (LDL) deposition and endothelial dysfunction trigger a local inflammatory response. As the disease progresses, persistent proinflammatory signaling promotes macrophage activation, foam cell formation, and fibrous cap destabilization. Notably, emerging evidence suggests that advanced AS is a consequence of excessive inflammation and is characterized by impaired resolution of inflammation, a mechanism that is still under active investigation [124].

During AS progression, an imbalance between proinflammatory and pro-resolving signals is increasingly recognized as a defining pathological feature [72]. Fredman et al. reported a marked reduction in the RvD1/LTB4 ratio within human vulnerable plaques, suggesting that impaired PRLM biosynthesis contributes to defective inflammation resolution. Similarly, plasma PRLM levels are markedly decreased in both high-fat diet–fed Ldlr −/− mice and patients with AS [125]. These findings have spurred growing interest in clarifying the role of PRLMs in plaque formation, inflammation regulation, and vascular homeostasis, as well as in assessing their potential as therapeutic targets beyond conventional lipid-lowering strategies.

Experimental studies have further demonstrated that PRLMs enhance plaque stability through several complementary mechanisms. They promote macrophage polarization toward the reparative M2 phenotype, suppress the local production of proinflammatory cytokines and ROS, and inhibit the activation of matrix metalloproteinases (MMPs) [126]. For example, exogenous administration of RvD1 in Ldlr −/− mice markedly reduced ROS generation and necrotic core expansion while simultaneously promoting fibrous cap formation and lowering collagenase levels [71]. Likewise, the delivery of RvD2 and MaR1 prevented AS progression by driving macrophage polarization toward a reparative phenotype and stimulating collagen synthesis in VSMCs [72]. In addition, RvE1 demonstrated synergistic effects with statins in several AS models, markedly reducing proinflammatory cytokine levels, inhibiting foam cell formation, and delaying vascular calcification. Finally, the administration of AT-LXA4 suppressed AS progression in ApoE −/− mice via the FPR2 receptor, underscoring the critical role of PRLM receptor pathways in regulating AS [127].

However, the protective effects of PRLMs depend on the activation of specific receptors, and accumulating evidence highlights the dual nature of receptor signaling. Under high-fat diet conditions, deletion of the RvE1 receptor ChemR23 markedly accelerates AS progression [128]. Similarly, ALX-FPR2 is expressed in macrophages, endothelial cells, and VSMCs within human atherosclerotic lesions; however, its functional consequences differ across models [129]. In some contexts, FPR2 deficiency reduces macrophage infiltration and attenuates lesion development, whereas in others, it lowers collagen content and increases plaque instability [130]. This “double-edged sword” effect highlights the importance of tailoring PRLM-based therapeutic strategies to the patient’s inflammatory status, receptor distribution, and local microenvironment.

PRLMs also exert state-dependent effects on neutrophil function. In healthy individuals, LXs enhance the oxidative burst of neutrophils, promoting efficient pathogen clearance. By contrast, in patients with AS, LXs suppress excessive ROS production and downregulate CD11b integrin activation, which limits neutrophil transendothelial migration and alleviates local inflammation [131]. This context-specific activity underscores the multifaceted role of PRLMs in immune homeostasis and highlights new opportunities for dynamic immune interventions and precision therapy.

PRLMs show considerable promise for the prevention and treatment of AS through diverse mechanisms, including immune cell function modulation, plaque stabilization, and vascular microenvironment improvement. However, their rapid metabolism, short half-lives, and heterogeneous receptor signaling remain major barriers to clinical translation. To address these challenges, future research should integrate multi-omics approaches to delineate the distinct functions of individual PRLM subtypes, develop precision delivery strategies targeting receptor–ligand interactions, and evaluate their efficacy and safety in early phase clinical trials. Such efforts will be critical for translating PRLMs from experimental studies into clinical practice.

#### Aneurysm

An aortic aneurysm is defined as focal dilation of the aorta and arises from pathological processes, including inflammatory cell infiltration, ECM degradation, VSMC apoptosis, and oxidative stress. Together, these mechanisms drive vascular remodeling, manifested as adventitial thickening and medial elastic fiber destruction [132]. Although abdominal aortic aneurysm (AAA) has traditionally been linked to AS, accumulating evidence indicates that these two conditions differ in their pathogenic mechanisms. AAA development is thought to begin with early infiltration of inflammatory cells into the adventitia, including macrophages, T and B lymphocytes, and neutrophils, which subsequently migrate into the media. In vitro studies have further demonstrated that activated macrophages secrete MMPs and proinflammatory cytokines, leading to ECM degradation and VSMC apoptosis. These processes gradually weaken the aortic wall, ultimately causing vascular dilation and an increased risk of rupture [133, 134].

In recent years, the role of inflammation in AAA pathogenesis has become increasingly evident, shifting the perspective from viewing the disease solely as a degenerative condition to recognizing it as a failure of inflammatory resolution. Researchers have interpreted AAA progression as an imbalance between proinflammatory signals and PRLMs. Despite this conceptual advance, clinical evidence directly linking PRLMs to patient outcomes is scarce. For instance, one study analyzing plasma lipid mediator profiles from patients undergoing AAA surgery identified two metabolic patterns: a proinflammatory profile characterized by elevated levels of LTs, prostaglandins, and their precursors; and a pro-resolving profile marked by increased levels of RvE1, RvE2, RvD1, MaR1, and their precursors (17-HDHA and 14-HDHA). However, this study did not assess the correlations between these lipid mediator profiles and AAA progression or postoperative recovery, underscoring a critical gap in translational research [135].

Supporting these observations, experimental studies have highlighted the receptor-dependent protective effects of PRLMs. Aortic tissues from patients undergoing elective open AAA surgery demonstrated markedly reduced mRNA expression of ALX-FPR2, the primary receptor for LXA4 and RvD1, in the adventitia compared with that of non-aneurysmal donors [136]. Consistent with these findings, ApoE −/− mice lacking the FPR2 receptor develop exaggerated aortic dilation following Ang II infusion, accompanied by increased MMP9 expression, reduced collagen deposition, elastic fiber fragmentation, and abnormal infiltration of neutrophils and macrophages [136]. Similar outcomes were observed in mice deficient in 12/15-LOX, a key enzyme for LXA4 and RvD1 biosynthesis, further supporting the protective role of endogenous PRLMs in AAA pathogenesis.

Functional studies have provided mechanistic insights into these protective effects. Multiple animal models have shown that RvD1 and RvD2 markedly inhibit AAA initiation and progression [137, 138]. In an Ang II–induced AAA model in ApoE −/− mice, RvD2 treatment suppressed proinflammatory mediators (MCP-1, IL-6, IL-1β, and RANTES) and MMP2/9 activity while upregulating the anti-inflammatory cytokine IL-10. These changes reduced the lesion size and improved vascular remodeling. Mechanistically, protection was primarily mediated by macrophage polarization toward the M2 reparative phenotype, whereas T-cell and neutrophil infiltration were largely unaffected. Similar protective effects of RvD1 and RvD2 were observed in an elastase perfusion–induced AAA model. RvD2 supplementation, even after AAA formation, continued to suppress progressive aortic dilation, suggesting its potential to delay disease progression [138].

PRLMs demonstrate substantial protective potential in AAA by suppressing proinflammatory signaling, modulating immune cell polarization, and fine-tuning the vascular immune microenvironment through receptor pathways such as ALX-FPR2 and LGR6. Nevertheless, most evidence comes from mouse models, and human clinical data remain limited, particularly regarding the dynamic monitoring of lipid mediator profiles and their prognostic relevance. Future research should integrate multi-omics approaches with precision delivery technologies to delineate the specific roles of different PRLM subtypes, identify therapeutic windows across disease stages, and advance early phase clinical trials. These efforts will be crucial for translating PRLM-based therapies into clinical practice and improving outcomes for patients with AAA.

### Metabolic diseases

Growing evidence indicates that inflammation is a central driver of metabolic diseases, particularly obesity and type 2 diabetes, and their associated complications [139]. In recent decades, studies have clarified how obesity-associated inflammation contributes to insulin resistance and impaired glucose tolerance. Adipose tissue is a metabolically active endocrine organ that secretes adipokines, cytokines, and chemokines that regulate energy homeostasis. However, when caloric intake surpasses the lipid storage capacity of white adipose tissue, excess lipids accumulate in non-adipose organs, such as the liver, skeletal muscle, kidney, and pancreas, initiating chronic local inflammation [140]. Such persistent proinflammatory signaling has been documented not only in the three classical insulin target tissues—adipose tissue, liver, and muscle—but also in the central nervous system (CNS) and gastrointestinal tract, highlighting the systemic nature of metabolic inflammation underlying obesity and diabetes.

Macrophages are the central regulators of this inflammatory milieu. In obese adipose tissue, proinflammatory M1 macrophages markedly increase, driving chronic inflammation and insulin resistance, whereas anti-inflammatory M2 macrophages contribute to tissue repair and resolution. PRLMs have emerged as promising regulators that can restore this balance. Ex vivo studies have demonstrated that PRLMs reduce adipose tissue inflammation, enhance the generation of anti-inflammatory lipid mediators, and promote macrophage polarization toward an M2-like phenotype [141]. For instance, Börgeson et al. reported that LXs suppress inflammation by shifting adipose tissue–resident macrophages from an M1 to a pro-resolving M2-like state [142]. However, enhancing endogenous LX levels remains challenging because the 12/15-LOX enzymes required for LX biosynthesis also drive the production of proinflammatory LTs and prostaglandins [143]. Additional in vitro studies revealed that overexpression of 15-LOX in mesenchymal stem cells increased LXA4 production and modulated the Th17/Treg balance toward an anti-inflammatory Treg phenotype [144]. Fredman et al. further showed that LXs alter the subcellular localization of 5-LOX, redirecting its activity from nuclear LTB4 synthesis to cytoplasmic LX production, suggesting that PRLMs may exert anti-inflammatory effects through spatial regulation [145].

Despite the recognized benefits of PUFAs for metabolic health, their ability to regulate adipose tissue inflammation through PRLM pathways remains uncertain. In a double-blind randomized trial, obese individuals who consumed fish oil concentrates for 12 weeks showed modest improvements in the adipose tissue expression of inflammation-related genes. However, ω−3–derived PRLM levels changed only minimally, and the intervention effects were more pronounced in lean individuals [146]. Consistently, in obese women undergoing bariatric surgery, a short-term low-calorie diet (2 weeks) markedly reduced the adipose tissue expression of the inflammatory markers CD45 and CCL2 [147]. Moreover, moderate weight loss enhanced the ability of neutrophils from patients with obesity to secrete RvE1 upon stimulation. Supporting these human findings, studies in Diversity Outbred mice demonstrated that RvE1 treatment improved gastric inhibitory polypeptide, insulin, glucagon, and leptin levels in a subset of obese mice, suggesting that therapeutic responses to PRLMs may vary depending on the metabolic status [148].

Besides LXs and RvE1, other PRLMs, such as RvD1 and MaRs (MaR1 and MaR2), play important roles in regulating metabolic inflammation. In co-culture systems of human adipocytes and macrophages, RvD1 markedly and dose-dependently reduced proinflammatory cytokine secretion by acting on macrophages rather than directly on adipocytes [149]. It further decreased macrophage adhesion and migration, promoted polarization toward a pro-resolving M2 phenotype, and enhanced phagocytic activity [62]. In parallel, MaR1 mitigates the inhibitory effects of TNF-α on glucose uptake and Akt phosphorylation in adipocytes while improving hyperglycemia and insulin resistance in diet-induced obese mice [150]. Moreover, both MaR1 and MaR2 contribute to the activation of BAT and the browning of white adipose tissue, increasing energy expenditure and promoting inflammation resolution [151, 152]. These mechanisms apply to type 2 diabetes, in which impaired insulin signaling and persistent low-grade inflammation accelerate disease progression and metabolic dysfunction.

Collectively, these findings highlight the multifaceted roles of PRLMs in modulating immune cell function, improving adipose tissue homeostasis, and restoring metabolic balance in obesity and type 2 diabetes. Nevertheless, most evidence has been derived from animal models and in vitro studies, with limited clinical validation. Future research should integrate multi-omics technologies with longitudinal clinical cohorts to define therapeutic windows and inter-individual variations in PRLMs responses, identify predictive biomarkers, and explore combinatorial strategies with established metabolic interventions, such as GLP-1 receptor agonists and SGLT2 inhibitors. Such efforts could accelerate the translation of PRLM-based therapies from experimental models to clinical practice and ultimately reshape the management of metabolic diseases.

### Neurodegenerative diseases (NDDs)

NDDs are a heterogeneous group of disorders characterized by progressive neuronal loss in the CNS and peripheral nervous system [153]. A defining feature of many NDDs is the misfolding and aggregation of proteins, which directly damage neurons and activate the microglia and astrocytes. This glial activation initiates neuroinflammation, accelerating neuronal dysfunction. While neuroinflammation was traditionally considered a secondary response to protein aggregation, accumulating evidence suggests that inflammatory processes often precede protein deposition and may even act as initiating events in neurodegenerative pathology [154].

In the early stages of the disease, neuroinflammation can play a protective role by activating immune responses that eliminate pathogens and cellular debris, preserving neural homeostasis [155]. However, as the disease progresses, prolonged overactivation of microglia and astrocytes drives the persistent release of proinflammatory mediators, such as IL-1β and TNF-α, creating a self-sustaining inflammatory microenvironment [154]. Emerging evidence indicates that chronic inflammation arises from excessive immune activation and impaired endogenous resolution mechanisms. A key contributor to this failure is the downregulation of PRLM expression. Clinical and experimental studies have consistently demonstrated that PRLM levels are markedly reduced in the cerebrospinal fluid and affected brain regions of patients with NDDs, with lower levels correlating with more severe cognitive decline. These findings highlight PRLMs as central regulators of disease progression [156].

Within the CNS, PRLMs exert neuroprotective effects via multiple complementary mechanisms. They suppress proinflammatory signaling pathways, such as NF-κB, reduce the release of inflammatory mediators, including IL-1β and TNF-α, and reprogram microglia and astrocytes to enhance the clearance of pathological proteins, such as Aβ and α-synuclein (α-syn). In addition, PRLMs regulate synaptic plasticity and activate neuroregenerative pathways, preserving neuronal function and structural integrity [157].

Together, these findings demonstrate that PRLMs play a pivotal role in linking unresolved neuroinflammation to the progression of the disease. Advancing research has shown that their discovery is driving a paradigm shift in the study of NDDs—from the traditional emphasis on “suppressing inflammation” to the active promotion of “inflammation resolution.” This conceptual transition not only deepens our understanding of disease pathogenesis but also creates new opportunities for clinical interventions. In the following sections, we systematically summarize the mechanisms, recent advances, and translational potential of PRLMs in NDDs, including Alzheimer’s disease (AD), Parkinson’s disease, Cerebral infarction (CI), and Vascular mild cognitive impairment (VaMCI). The aim is to establish both a theoretical framework and practical guidance for developing precision therapies.

#### Alzheimer’s disease (AD)

AD, the most common cause of dementia, is pathologically characterized by the extracellular deposition of amyloid-β (Aβ) in diffuse and neuritic plaques, together with neurofibrillary tangles and neuropil threads formed by aggregates of hyperphosphorylated tau protein in degenerating neurons [158, 159]. Besides these proteinopathies, increasing evidence has identified neuroinflammation as a critical hallmark of AD. Patients with AD consistently exhibit elevated levels of proinflammatory cytokines in both plasma and brain tissue, accompanied by profound impairment of inflammation-resolving pathways [156]. These observations suggest that the dysregulation of inflammatory resolution may play a central role in disease progression.

Building on this framework, multiple clinical studies have highlighted the regulatory roles of PRLMs in AD pathogenesis. In the entorhinal cortex of patients with AD, MaR1 levels are markedly lower than those in age-matched controls, whereas the concentrations of the proinflammatory mediator prostaglandin D2 (PGD2) are elevated. RvD4 levels further demonstrate an inverse correlation with biomarkers of neurofibrillary tangles and a positive correlation with cognitive performance [160]. Consistent with these findings, a cerebrospinal fluid lipidomic study revealed that PRLMs, including RvD4, RvD1, PD1, MaR1, and RvE4, were markedly reduced in patients with AD and mild cognitive impairment (MCI) compared with those of controls. Within this lipid mediator profile, RvD1 is negatively associated with phosphorylated tau (p-tau) concentrations, whereas RvD4 is inversely related to the severity of neurofibrillary tangles [161]. Collectively, these clinical observations suggest that PRLMs may serve not only as potential biomarkers for early AD diagnosis but also as modulators of disease progression.

Mechanistic studies have further supported these clinical associations. The neuroprotective effects of PRLMs are partly mediated by their regulation of pathological protein metabolism and broader influence on inflammatory networks. MaR1 enhances neuronal survival and promotes microglial phagocytosis of Aβ by activating PPAR-γ signaling [58]. Similarly, exogenous administration of RvE1 and LXA4 increases microglial clearance of Aβ and improves cognitive function in AD mouse models [162]. Besides protein clearance, PRLMs regulate the neuroinflammatory milieu. In AD, chronically overactivated microglia release proinflammatory cytokines, such as IL-1β and TNF-α, generating a self-perpetuating cycle of inflammation and neuronal injury. PRLMs disrupt this cycle by suppressing NF-κB signaling, reducing proinflammatory mediator production, and promoting microglial polarization toward an anti-inflammatory phenotype [163]. Furthermore, PRLMs activate the PI3K/Akt pathway, which prevents neuronal apoptosis, maintains synaptic plasticity, and supports neural network integrity [164].

Taken together, the current evidence indicates that PRLMs confer neuroprotection in AD through complementary mechanisms, including suppression of neuroinflammation, enhancement of pathological protein clearance, reprogramming of glial phenotypes, and direct preservation of neuronal survival and synaptic function. These findings highlight PRLMs as promising candidates not only for early diagnosis and disease staging but also as molecular targets for developing novel therapeutic strategies aimed at promoting inflammation resolution. As research advances, PRLMs may provide a conceptual and translational bridge between understanding AD pathogenesis and developing precision-based interventions, laying the groundwork for future therapeutic breakthroughs.

#### Parkinson’s disease

Parkinson’s disease, the second most common neurodegenerative disorder after AD, is pathologically characterized by neuronal inclusions known as Lewy bodies and neurites, along with the progressive loss of dopaminergic (DA) neurons in the substantia nigra pars compacta (SNpc). Lewy bodies primarily comprise aggregated and misfolded α-syn. Abnormal α-syn accumulation triggers microglial activation and proliferation within the SNpc, promoting the release of inflammatory cytokines, which may occur before DA neuron loss [165]. Persistent neuroinflammation, T-cell infiltration, and glial activation are consistently observed in both patients with parkinson’s disease and experimental models, where they play critical roles in DA neuron degeneration [166]. The particular vulnerability of DA neurons to inflammatory injury further highlights the central contribution of microglia-mediated inflammation to pparkinson’s disease progression [167].

Insights into the inflammatory basis of parkinson’s disease have prompted increasing interest in specialized PRLMs as potential modulators of disease progression. Paraskevi Krashia et al. reported that RvD1 levels were markedly reduced in an α-syn–induced parkinson’s disease model. Early and sustained RvD1 administration in Syn rats effectively attenuated neuroinflammation, restored DA neurotransmission, and prevented neuronal deficits and motor impairments [59]. Subsequent studies have shown that the neuroprotective effects of RvD1 are mediated through the activation of the ALX/FPR2, which is expressed in both glial cells and neurons. At the molecular level, RvD1 protects against inflammation-induced neuronal death by regulating downstream signaling via miR-146b and miR-219a-1 [168].

Building on these findings, recent studies have emphasized the spatiotemporal specificity of PRLMs activity in parkinson’s disease. In the early stages of the disease, PRLMs, such as RvD1, help preserve DA neuron survival and function by suppressing microglial overactivation and limiting the release of proinflammatory cytokines. However, as parkinson’s disease progresses, the neuroinflammatory microenvironment becomes increasingly complex. Immune cell imbalance, oxidative stress, and neurodegenerative pathology interact to diminish the efficacy of PRLM-based interventions. Therefore, future studies should aim to map the dynamic changes in PRLMs signaling pathways across different stages of parkinson’s disease and explore their potential synergy with anti-inflammatory drugs, neuroprotective strategies, or α-syn–targeted therapies. These efforts will provide a stronger theoretical foundation for precision interventions and facilitate clinical translation.

#### Cerebral infarction (CI)

CI results from impaired cerebral circulation, leading to local hypoxia, ischemia, and tissue necrosis, ultimately causing neurological dysfunction. AS is the principal risk factor for acute CI, and unstable or ruptured plaques can obstruct cerebral vessels, reduce perfusion, and trigger ischemic injury [169]. During ischemia–reperfusion (I/R), excessive production of ROS, release of proinflammatory cytokines, and aberrant immune cell infiltration act synergistically to accelerate neuronal death and tissue damage. Consequently, therapeutic strategies targeting the resolution of inflammation, oxidative stress, and vascular homeostasis have become central areas of ongoing research.

Within this pathological context, specialized PRLMs have gained increasing attention for their dual ability to promote inflammation resolution and provide neuroprotection. Early studies have demonstrated that the administration of a DHA-enriched triacylglycerol emulsion markedly reduces brain injury in neonatal hypoxic–ischemic models, underscoring the essential role of lipid metabolites in neuroprotection [170, 171]. Building on these findings, subsequent animal studies have shown that exogenous RvD1 enhances atherosclerotic plaque stability, increases fibrous cap thickness, and reduces oxidative stress and necrotic core size, indirectly modulating stroke-related risk factors [71]. In middle cerebral artery occlusion (MCAO) models, treatment with exogenous LXA4 provides robust neuroprotection against IRI, markedly decreasing infarct volume and improving neurological outcomes [172, 173]. Similarly, RvD2 levels were markedly reduced in MCAO animals, whereas intraperitoneal administration of exogenous RvD2 effectively reversed ischemic injury, as evidenced by reduced infarct size, attenuated brain edema, dampened inflammatory responses, and preserved neuronal function [174]. Mechanistic investigations further revealed that RvD2 exerts its effects by binding to the GPR18, which inhibits ERK1/2–iNOS signaling and mitigates both inflammation and oxidative stress [175].

The DHA-derived mediator, NPD1, has also demonstrated strong efficacy in multiple experimental studies. NPD1 not only reduces infarct size and improves neurological recovery but also attenuates inflammation by inhibiting NF-κB activation, downregulating COX-2 expression, and limiting polymorphonuclear neutrophil (PMN) infiltration [176]. In a rat I/R model, intracerebroventricular administration of NPD1 restricted calpain-mediated proteolysis of TRPC6 and subsequently activated cAMP response element-binding protein (CREB) through the Ras/mitogen-activated protein kinase (MEK)/ERK pathway, resulting in smaller infarct volumes and improved functional outcomes [177].

Taken together, the current evidence highlights the multifaceted neuroprotective actions of PRLMs, including the suppression of oxidative stress, stabilization of vascular lesions, regulation of immune cell polarization, and inhibition of proinflammatory signaling pathways. These findings suggest that PRLMs are promising therapeutic candidates for CI. However, their spatiotemporal specificity across different stages of disease progression remains poorly understood, and effective clinical translation will require systematic research and the development of precision-targeted strategies.

#### Vascular mild cognitive impairment (VaMCI)

VaMCI is an early clinical stage of cognitive decline associated with cerebrovascular injury and serves as a transitional state between normal cognition and vascular dementia (VaD). Patients with VaMCI commonly present with higher-order cognitive impairments, including deficits in executive functioning, slowed information processing, and memory decline. The pathogenesis of VaMCI is complex and multifactorial, encompassing neuronal apoptosis, oxidative stress, impaired synaptic plasticity, disrupted neural networks, and bidirectional dysregulation between the CNS and peripheral inflammation [178, 179]. These pathological processes are closely associated with AS, cerebral small vessel disease, and blood–brain barrier (BBB) dysfunction, positioning VaMCI as an important disease model for investigating cerebrovascular–neural interactions.

At the molecular level, PRLMs have attracted increasing interest because of their potential to modulate neuroinflammation, restore metabolic balance, and maintain neural homeostasis. Clinical studies have demonstrated that supplementation with marine-derived PUFAs and antioxidants in VaMCI patients markedly elevated RvD1 levels in macrophages derived from peripheral blood mononuclear cells (PBMCs) [180, 181]. This suggests that PRLMs may improve the neuroinflammatory microenvironment by modulating peripheral immune cell activity. Another study showed that ω−3 fatty acids activate the cytoprotective protein kinase R–like endoplasmic reticulum kinase (PERK) pathway, alleviating endoplasmic reticulum stress, facilitating the clearance of misfolded proteins, and enhancing Aβ phagocytosis [182]. These mechanisms parallel those observed in AD, implying that VaMCI may share overlapping inflammatory and metabolic pathways with AD at the molecular level.

VaMCI is regarded as an early stage of AD, characterized by dual pathological features of cerebrovascular lesions and neurodegeneration. PRLMs may exert multifaceted neuroprotective effects in VaMCI by suppressing oxidative stress, reducing neuronal apoptosis, enhancing synaptic plasticity, promoting resolution of inflammation, and maintaining metabolic homeostasis in the brain. Collectively, these actions not only provide novel strategies for early intervention in VaMCI but also offer potential breakthroughs for elucidating the pathological links between vascular cognitive impairment and AD and for advancing targeted therapeutic approaches.

### Respiratory diseases

#### Asthma

Asthma is a heterogeneous disorder characterized by chronic airway inflammation driven by immune imbalance, airway hyperresponsiveness, and complex multifactorial interactions. In patients with asthma, AA is metabolized by 5-LOX and leukotriene C4 synthase to generate cysLTs, including leukotriene C4 (LTC4), LTD4, and LTE4 [183]. These mediators induce airway smooth muscle contraction, bronchospasm, tissue edema, and mucus hypersecretion, playing a central role in the pathogenesis of the disease [184]. Excessive activation of eosinophils further amplifies this process by releasing large amounts of inflammatory cysLTs, which fuel downstream allergic responses [185]. Clinical studies have identified urinary cysLT levels as potential biomarkers of type 2 asthma inflammation [186]. However, although cysLTR1 antagonists, such as montelukast, can partially alleviate symptoms by blocking receptor activity, their efficacy varies markedly among individuals, indicating that additional inflammatory pathways beyond cysLTs remain insufficiently understood [187].

Against this background, growing attention has been directed toward the role of specialized PRLMs in asthma, particularly their functions in promoting inflammation resolution and tissue repair. A novel subgroup of PRLMs from the MaR family, MCTRs, has been identified as a bioactive metabolite derived from the DHA metabolic pathway [30]. In healthy human lung tissue, MCTR1, MCTR2, and MCTR3 are detectable, with MCTR3 showing a strong inhibitory effect on LTD4-induced airway constriction in an ex vivo bronchospasm model. Montelukast only partially blocked the effects of MCTR3, suggesting that MCTRs may counteract LT-driven responses through interactions with cysLT receptors.

In vivo evidence further supports these findings. In an ovalbumin-induced mouse model of allergic airway inflammation, MCTRs played a pivotal role in the resolution phase of asthma. Administration of MCTRs at the peak of allergic inflammation markedly reduced airway hyperresponsiveness, eosinophil infiltration, and serum IgE levels, while improving airway barrier integrity by alleviating pulmonary edema. These results indicate that PRLMs not only regulate inflammatory resolution but may also influence airway remodeling, offering new molecular targets for refractory asthma [188].

Collectively, PRLMs demonstrate substantial therapeutic potential in asthma by blocking cysLT-driven allergic inflammation, enhancing airway barrier function, and promoting resolution processes [185]. Looking forward, the development of drugs and precision-targeted strategies targeting PRLM signaling pathways may help overcome the limitations of current anti-inflammatory therapies and provide innovative treatment options for complex and severe asthma.

#### Cystic fibrosis (CF)

Among pediatric respiratory diseases, CF is the most marked chronic inflammatory disorder after asthma [189]. CF is an inherited multisystem disease, with the most severe pathological manifestations occurring in the lungs and gastrointestinal tract. The root cause of CF is mutations in the cystic fibrosis transmembrane conductance regulator (CFTR) gene, which impair epithelial chloride and bicarbonate secretion. CF is characterized by neutrophil-driven inflammation, impaired airway mucus clearance, recurrent bacterial colonization, and progressive structural airway damage, ultimately culminates in bronchiectasis [189].

Early clinical biopsy studies revealed that the AA/DHA ratio in airway epithelial tissue was markedly elevated in patients with CF, suggesting that lipid metabolic imbalance is a central pathological feature and sparking interest in DHA supplementation [190]. Subsequent analyses of AA metabolites demonstrated that LTB4 levels were markedly increased, whereas LXA4 levels were markedly decreased in patients with CF [191]. As LTB4 is a potent neutrophil chemoattractant and LXA4 is a key pro-resolving mediator, this imbalance indicates an impaired resolution of inflammation. In patients with CF and chronic Pseudomonas aeruginosa infection, bacterial enzymes can directly disrupt LXA4 synthesis and activity, underscoring the profound influence of host–pathogen interactions on the lipid mediator profile of CF [192].

Recent mechanistic studies have increasingly highlighted the anti-inflammatory and immunomodulatory functions of RvD1 in CF. In animal models, RvD1 markedly reduced the pulmonary bacterial burden and neutrophil infiltration in mice with chronic Pseudomonas infection [193]. Complementary in vitro experiments using neutrophils and macrophages derived from patients with CF further showed that RvD1 enhanced bacterial phagocytosis while suppressing the production of inflammatory cytokines, including IL-8 and IL-6. These experimental findings align with clinical observations demonstrating that sputum RvD1 levels in patients with CF are inversely correlated with inflammation severity [194]. Collectively, this body of evidence suggests that PRLMs regulate CF-related inflammation by modulating neutrophil function and restoring the balance between proinflammatory and pro-resolving mediators.

Clinical studies on DHA supplementation have yielded inconsistent results. While some small-scale studies have reported improvements in the AA/DHA ratio and reductions in LTB4 levels, most multicenter, double-blind, placebo-controlled trials have failed to demonstrate marked benefits of DHA supplementation on lung function, clinical outcomes, or inflammatory markers [195]. Most investigations have focused on DHA itself rather than its downstream PRLMs derivatives. For example, RvD1 and related mediators act directly within the CF inflammatory network, suggesting that they may provide more targeted and effective therapeutic potential than DHA supplementation alone.

CF is defined as an imbalance between proinflammatory and PRLMs, characterized by excessive LTB4 accumulation and insufficient PRLM levels, such as LXA4 and RvD1. This dysregulation provides a strong mechanistic rationale for precision immunomodulatory intervention. Future studies should prioritize elucidating the pharmacological actions and clinical relevance of individual PRLMs, particularly RvD1 and LXA4, in CF. Special emphasis should also be placed on evaluating their potential in combination with CFTR modulators and antimicrobial therapies, which may open new therapeutic avenues for effective disease management.

#### Acute respiratory distress syndrome (ARDS)

ARDS is a life-threatening condition characterized by hypoxemia, reduced lung compliance, and diffuse alveolar injury, largely driven by uncontrolled inflammation within the alveolar space [196]. Its etiology is multifactorial and includes respiratory infections, trauma, and systemic inflammatory responses. Current management primarily relies on lung-protective ventilation with low tidal volume and strict fluid control. However, the absence of effective targeted pharmacological therapies contributes to the persistently high mortality. Consequently, the precise control of excessive inflammation while simultaneously promoting its resolution remains a major challenge in advancing ARDS treatment.

In recent years, increasing attention has been directed toward the roles of PRLMs and their associated signaling pathways in ARDS. PRLMs are central regulators of inflammation resolution, immune homeostasis, and tissue repair, making them promising therapeutic targets. A meta-analysis of 12 randomized controlled trials demonstrated that ω−3 PUFA supplementation improved oxygenation and showed a trend toward reducing intensive care unit (ICU) length of stay and duration of mechanical ventilation. However, no consistent improvements were observed in mortality or secondary infection risk [197]. Similarly, the Omega trial, a randomized placebo-controlled study, failed to demonstrate the clear benefits of PUFA-based supplementation in ARDS [198]. These findings suggest that ω−3 supplementation alone may be insufficient for meaningful clinical benefits and highlight the need to investigate the direct immunoregulatory actions of PRLMs.

Emerging evidence further supports the association between PRLM deficiency and adverse clinical outcomes in ARDS. A plasma-based study in patients with ARDS showed that low PRLM levels were markedly associated with prolonged mechanical ventilation and extended ICU stays [199]. As ARDS frequently develops secondary to sepsis, blood analyses in patients with sepsis revealed that stronger RvD1-induced enhancement of neutrophil phagocytosis correlated with markedly reduced requirements for mechanical ventilation [200]. Collectively, these findings suggest that early activation of PRLM pathways may help restrain excessive immune activation and reduce both the incidence and severity of ARDS.

The role of PRLMs has also been investigated in COVID-19–related ARDS. A study of 33 patients with severe COVID-19 found markedly elevated levels of LXA4 and RvDs in bronchoalveolar lavage fluid (BAL) [201]. However, these patients simultaneously exhibit increased concentrations of proinflammatory lipid mediators, including thromboxane B2, prostaglandins, and LTs, indicative of a lipid mediator storm characterized by an imbalance between proinflammatory and pro-resolving signals. Plasma analyses further revealed that patients with milder disease who did not require noninvasive ventilation or high-flow oxygen support had markedly higher PRLM levels, particularly protectin conjugate in tissue regeneration 3 (PCTR3) and maresin conjugate in tissue regeneration 3 (MCTR3), than those with more severe illness [202]. Consistently, another study reported that patients hospitalized with COVID-19 who avoided ICU admission had higher serum RvE3 levels, reinforcing the association between PRLMs abundance and disease severity [203].

In patients with severe COVID-19, lower plasma PRLM levels among those requiring mechanical ventilation were associated with higher mortality, even when the overall plasma concentrations were elevated. PRLM levels were also markedly reduced in high-risk populations, such as the older adults and obese, which may partly explain their increased susceptibility to severe disease [204]. PRLM concentrations can be modulated by pharmacological interventions. For example, dexamethasone, one of the first effective treatments for COVID-19, improved survival in critically ill patients, activated PRLM biosynthetic pathways, and increased plasma concentrations [205]. In parallel, an ongoing clinical trial is evaluating the therapeutic potential of parenteral ω−3 PUFA supplementation in patients hospitalized with COVID-19 [206].

Taken together, the current evidence indicates that PRLMs are central regulators of inflammation in ARDS and COVID-19–related lung injury. Nonetheless, critical questions remain regarding the optimal timing and dosing of PRLMs or precursor supplementation, as well as how to individualize interventions based on the inflammatory status. Addressing these uncertainties will require large-scale multicenter clinical trials. Such studies will not only clarify the precise mechanisms of PRLMs in ARDS pathophysiology but also provide a foundation for developing novel therapeutic strategies.

#### Smoking and environment-related lung injury

Smoking is the most common environmental factor contributing to lung injury, and the respiratory tract is also chronically exposed to particulates, dust, smoke, and other inhaled toxins. These exposures substantially increase the global burden of pulmonary diseases and promote the development of chronic airway inflammation [207]. Among smoking-related conditions, chronic obstructive pulmonary disease (COPD) is the most representative example. Analyses of sputum and BAL from patients with COPD reveal a marked imbalance between proinflammatory and PRLMs, with markedly elevated levels of LTs and markedly reduced levels of PRLMs [208].

Building on these findings, human and experimental studies further support the role of disrupted resolution pathways in smoking-related injuries. A plasma-based study showed that smokers, compared with non-smokers, exhibited elevated levels of inflammatory cytokines (IL-6, IL-8, and TNF) and markedly reduced concentrations of RvE1 [209]. Similarly, the use of electronic cigarettes increased plasma inflammatory markers and was associated with decreased levels of RvD1 and RvD2 [210]. In murine models, tobacco smoke exposure impairs immune responses to Haemophilus influenzae, a common pathogen responsible for acute COPD exacerbations. Treatment with aspirin-triggered RvD1 (AT-RvD1) reduced inflammatory cytokine levels, diminished inflammatory cell infiltration in BAL, and restored functional immune responses in the lung [211]. Taken together, these findings suggest that restoring PRLM levels or activity may help mitigate smoking-induced lung injury and reduce the risk of acute COPD exacerbations.

Beyond smoking, other environmental exposures also influence PRLM metabolism and the inflammatory balance. Animal studies have demonstrated that ozone exposure triggers pulmonary inflammation, accompanied by reduced levels of PRLM precursors (14-HDHA and 17-HDHA) and protectin DX (PDX) [212]. This effect was observed only in male mice, suggesting that sex differences may influence ozone-related inflammatory responses [213]. Organic dust exposure has also been shown to disrupt PRLM metabolism. In a mouse model, animals exposed to organic agricultural dust and fed a DHA-enriched diet exhibited increased levels of RvD1 and RvD2 and decreased levels of AA in BAL compared with those on a standard diet [214, 215]. Consistently, a study on engineered carbon nanoparticles found that during the early phase of exposure (days 1–3), mice developed acute neutrophil-driven inflammation, characterized by elevated LTB4 and PGE2 levels. However, by day 7, RvD1 and RvE1 levels were markedly increased, highlighting the time-dependent role of PRLMs in driving resolution during the later stages of lung injury [214].

Collectively, current evidence indicates that smoking and environmental exposures suppress the synthesis and function of PRLMs and disrupt pro-resolving signaling pathways, perpetuating inflammation and accelerating the progression of chronic lung disease [215]. However, the immunomodulatory benefits of PRLM supplementation or upregulation in long-term smokers remain insufficiently validated in clinical studies, partly because of challenges in study design and population heterogeneity. Therefore, future research should aim to characterize the dynamic changes in PRLM metabolism under different environmental conditions and elucidate their mechanistic roles in chronic lung injury. Moreover, evaluating the feasibility of restoring immune balance through supplementation with ω−3 PUFAs or exogenous PRLMs may provide new therapeutic strategies for the prevention and treatment of smoking- and environment-related lung diseases.

### Tumors and the immune microenvironment

Cancer is a major global public health burden, characterized by high incidence and mortality, and arises from the interplay between genetic and environmental factors [216]. Beyond genetic mutations and aberrant cellular signaling, accumulating evidence shows that chronic inflammation is a critical driver of cancer initiation and progression, with nearly 25% of tumors believed to originate from persistent tissue inflammation [217]. This recognition has prompted researchers to re-evaluate the roles of social, environmental, and lifestyle factors in inflammation-driven tumorigenesis. For example, smoking, dietary habits, and microbial dysbiosis can induce chronic low-grade systemic inflammation, which not only contributes to cardiovascular diseases, diabetes, respiratory disorders, and NDDs but also increases the risk of cancer [218, 219]. As early as 1828, Jean Marjolin noted a potential link between inflammation and malignancy, and in 1863, Virchow proposed that cancer could arise from the persistent proliferation of damaged cells under chronic irritation [220]. Today, inflammation, particularly sustained innate immune–mediated inflammation, is widely accepted as a causal factor in the pathogenesis of numerous cancers.

Inflammation, as part of the body’s defense response, is protective during the acute phase, helping to eliminate exogenous pathogens, such as bacteria and viruses, as well as endogenous damaging factors, such as necrotic cells and toxic metabolites [221]. However, when inflammation is dysregulated and persists in a chronic state, it can profoundly alter the tissue microenvironment. These changes promote DNA damage, uncontrolled cell proliferation, and tumor immune evasion, creating conditions conducive to tumor development. Epidemiological evidence reinforces this connection: patients with ulcerative colitis (UC) have a 4–tenfold higher risk of developing colorectal cancer (CRC) compared with the general population [222]. Likewise, chronic prostatitis has been linked to prostate cancer and pelvic inflammatory disease to ovarian cancer, highlighting the carcinogenic potential of chronic inflammation across different organ systems.

Building on these observations, increasing attention has been paid to how chronic inflammation shapes the tumor immune microenvironment. Persistent inflammation disrupts local cytokine networks and immune cell composition and alters the functions of key components, including tumor-associated macrophages (TAMs), myeloid-derived suppressor cells (MDSCs), and Tregs. Collectively, these changes foster an immunosuppressive milieu that weakens the antitumor response. Against this backdrop, elucidating the mechanisms of inflammation resolution, particularly the role of PRLMs in restoring immune balance and promoting antitumor immunity, has emerged as a promising focus in cancer research. The following section examines alterations in PRLM expression within tumors and their therapeutic implications, offering new insights into the interplay between tumor immune evasion and inflammation regulation and establishing a conceptual basis for precision interventions in inflammation-associated cancers.

#### PRLMs and tumor angiogenesis

Tumor growth and distant metastasis critically depend on neovascularization, which not only supplies oxygen and nutrients but also provides a route for tumor cells to evade immune surveillance. Although anti-angiogenic therapies targeting vascular endothelial growth factor (VEGF) signaling have achieved clinical success, their benefits are often transient owing to adaptive resistance and adverse effects. This limitation has driven interest in PRLMs as alternative endogenous regulators of tumor angiogenesis. Emerging evidence suggests that PRLMs suppress pathological vessel formation through diverse molecular pathways, offering a complementary and potentially more sustainable strategy for tumor control [222, 223].

Among these mediators, LXA4 has been extensively studied. By binding to its receptor ALXR, LXA4 inhibits VEGFR phosphorylation and reduces the synthesis of proangiogenic factors, such as VEGF-C. LXA4 also suppresses the production of inflammatory mediators, including PGE2, LTB4, IL-6, and IL-8, in human Kaposi’s sarcoma cells [224]. Furthermore, LXA4 downregulates HIF-1α expression, limiting hypoxia-driven angiogenesis and restraining tumor progression [225]. These findings underscore the ability of LXA4 to simultaneously modulate angiogenic and inflammatory pathways.

Besides LXA4, other PRLMs, such as RvD1 and LXB4, have demonstrated anti-angiogenic activity in gastric cancer cell lines. Both suppress the signal transducer and activator of transcription 3 (STAT3) signaling pathway, reducing proangiogenic gene expression and VEGF-A release. Formyl peptide receptor 1 (FPR1) mediates these effects. Inhibition of FPR1 has been reported to enhance tumor angiogenesis while decreasing the activity of PRLM-synthesizing enzymes, receptors, and associated signaling components [226]. These results suggest that the PRLM–FPR1 axis functions as an endogenous brake on pathological angiogenesis. In addition, RvE1 has demonstrated anti-angiogenic potential, although direct evidence in tumor models remains limited. In a corneal neovascularization model, RvE1 inhibited vessel formation by binding to the ChemR23 (also known as ChemR21) [227]. Although this mechanism has not yet been systematically validated in tumors, it provides a theoretical framework for exploring RvE1’s role in maintaining vascular homeostasis within the tumor microenvironment (TME).

In summary, PRLMs target multiple angiogenic pathways and act at the interface of inflammation and vascular regulation, offering a new dimension to anti-angiogenic cancer therapy. Future research should investigate their integration with existing anti-angiogenic drugs, ability to overcome resistance, and translational potential in precision oncology. By bridging vascular biology and immunoregulation, PRLMs may help redefine strategies for durable tumor control.

#### PRLMs and tumor immunoregulation

Besides their established role in regulating tumor angiogenesis, PRLMs shape tumor initiation and progression through diverse immunoregulatory mechanisms. Within the TME, immune cells can undergo functional reprogramming, shifting from antitumor effectors to proinflammatory phenotypes that sustain tumor development under specific signaling cues. Tumor cells evade immune surveillance and modulate the surrounding immune milieu, promoting chronic inflammation and immune tolerance, which collectively support tumor growth and metastasis [228, 229]. Against this backdrop, PRLMs have emerged as critical modulators capable of restoring immune cell tumor-clearing functions by reshaping their activities within the TME, interfering with tumor evolution [230].

At the innate immunity level, PRLMs attenuate persistent inflammation by suppressing neutrophil and PMN chemotaxis, migration, and proinflammatory cytokine release. They also limit the generation of ROS and other toxic molecules, reducing DNA damage, genomic instability, and the associated risk of mutagenesis. In parallel, PRLMs enhance the phagocytic and cytotoxic activities of PMNs, conferring direct antitumor effects [231]. These actions extend beyond innate responses to influence adaptive immunity, highlighting the broad impact of PRLMs on immune regulation in the TME.

PRLMs have bidirectional effects on macrophage regulation. For instance, an ATL-1 analog was shown to inhibit the proliferation of monocyte precursors of TAMs, reducing TAM numbers, delaying tumor progression, and improving host survival [232]. Conversely, LXA4 has been reported to promote monocyte migration and polarization toward the M2 phenotype, maintaining an anti-inflammatory profile and enhancing phagocytosis, which may compromise antitumor efficacy [233]. Moreover, PRLMs activate the PI3K/Akt pathway to block apoptosis, upregulate Bcl-2 expression, stabilize mitochondrial function, and reduce ROS production, strengthening the anti-apoptotic and antioxidant properties of immune cells in the TME [234].

Within adaptive immunity, PRLMs regulate the functions of T, B, and natural killer (NK) cells. Preclinical studies have indicated that RvD1 preserves NK cell cytotoxicity, enhancing their ability to eliminate tumor cells. By contrast, LXA4 promotes the differentiation of Tregs, which may contribute to the establishment of early cancer immune tolerance [235]. LXA4 exerts the opposite effect on regulatory B cells (Bregs) by suppressing IL-10 production, restricting tumor growth [236]. Collectively, these findings underscore the multifaceted role of PRLMs in shaping both innate and adaptive immune responses, establishing their importance in reprogramming the immune landscape of the TME.

#### PRLMs and precancerous lesions

The inflammation-driven microenvironment is a critical determinant of precancerous lesion initiation. Chronic inflammation induces tissue damage, genetic mutations, and cellular dysfunction, fostering the emergence of precancerous states in high-risk organs [237]. These pathological changes not only precede the formation of invasive tumors but also represent key intervention points for secondary cancer prevention [238]. Within this framework, PRLMs, owing to their capacity to promote the resolution of inflammation, have been proposed as protective factors that may slow or prevent the pathological progression of precancerous lesions [239].

One important mechanism linking chronic inflammation to malignant transformation is epithelial–mesenchymal transition (EMT). EMT is characterized by the loss of intercellular junctions, downregulation of E-cadherin, and upregulation of mesenchymal markers such as vimentin, changes that confer migratory and invasive properties to cells [240]. Evidence indicates that PRLMs can inhibit both the initiation and progression of EMT via receptor-mediated pathways. For example, Zong et al. demonstrated that LXA4 reversed the EMT phenotype in pancreatic cancer cells by binding to the FPRL1 receptor, reducing local invasion and distant metastasis [241]. Similarly, RvD1, acting through FPR2, downregulated FOXM1 and COMP expression, blocked cancer-associated fibroblast (CAF)-induced EMT in hepatocytes, and markedly diminished cellular invasiveness [242]. Collectively, these findings suggest that PRLMs may restrain the transition of precancerous cells into invasive phenotypes by targeting multiple EMT signaling pathways.

Beyond EMT regulation, PRLMs also exhibit preventive potential through metabolic control in the early stages of tumorigenesis. A study examining the regulatory mechanisms of 15-lipoxygenase-1 (15-LOX-1), a key PRLM-synthesizing enzyme, found that its inhibition promoted the development of colitis-associated cancer (CAC) by activating the IL-6/STAT3 signaling pathway [243]. These results indicate that impaired PRLM synthesis or functional deficiency may accelerate the progression of precancerous lesions, underscoring PRLM metabolic regulation as a promising strategy for secondary cancer prevention.

Further support for the protective role of PRLMs comes from studies on precancerous liver lesions. In chronic hepatitis models, RvD1 and RvE1 have demonstrated marked protective effects. In the concanavalin A (ConA)-induced autoimmune hepatitis model, pretreatment with RvD1 or RvE1 markedly reduced the release of TNF-α, IFN-γ, IL-2, IL-1β, and IL-6, while simultaneously suppressing CD4⁺ and CD8⁺ T-cell infiltration through the inhibition of NF-κB and AP-1 signaling [244]. Likewise, PD1 and MaR1 alleviate liver injury and delay the progression from chronic inflammation to hepatocellular carcinoma by blocking the CX3CL1/CX3CR1 axis and reducing ROS production [245, 246]. Together, these findings highlight the multifaceted roles of PRLMs in modulating immune inflammation, maintaining tissue homeostasis, and preventing precancerous lesion progression. Looking ahead, further elucidation of the mechanistic roles of PRLMs in precancerous lesions may not only deepen our understanding of inflammation-driven carcinogenesis but also pave the way for their clinical translation as precision interventions aimed at halting malignant progression.

### Autoimmune diseases

Dysregulated and excessive immune activation is a fundamental pathogenic mechanism underlying autoimmune diseases. Precise control of inflammatory responses is required to prevent disease progression and tissue injury. Once this regulatory balance fails, inflammation may amplify in a feed-forward manner, driving a cascade of pathological events [247, 248]. Persistent leukocyte activation exemplifies this dysregulation and is observed in multiple autoimmune disorders, including rheumatoid arthritis (RA), multiple sclerosis (MS), systemic lupus erythematosus (SLE), inflammatory bowel disease (IBD), psoriasis (PSO), type 1 diabetes mellitus (T1DM), and Sjögren’s syndrome (SS). Current clinical management primarily relies on immunosuppression therapy. Broad-acting immunosuppressive drugs, such as glucocorticoids, methotrexate, tacrolimus, and cyclophosphamide, remain the first-line treatments for RA, MS, and SLE. While these agents effectively dampen excessive inflammation and improve symptoms, they also compromise systemic immune competence, leading to increased susceptibility to infections, malignancies, and long-term treatment-associated complications [249].

To overcome these limitations, therapeutic development has increasingly shifted toward targeted immunomodulation strategies, including molecular- and cell-specific agents, such as B cell–depleting monoclonal antibodies. However, substantial safety concerns persist; for example, prolonged B cell depletion may induce JC virus reactivation, leading to progressive multifocal leukoencephalopathy, an often fatal demyelinating condition [250]. Together, these challenges underscore the unmet clinical need for therapeutic approaches capable of resolving pathological inflammation while preserving host defense mechanisms and minimizing immune-related toxicity. Emerging resolution-based strategies, particularly those that promote endogenous inflammation-resolving pathways rather than broadly suppressing immunity, may offer a more precise and safer therapeutic paradigm for the management of autoimmune diseases.

#### Rheumatoid arthritis (RA)

RA is a common chronic autoimmune inflammatory disorder that affects approximately 1% of the global population [251]. A defining pathological feature of RA is the substantial infiltration of immune cells into the synovial membrane. Neutrophils are typically the first responders; they phagocytose pathogens and release inflammatory mediators that subsequently recruit monocytes, T cells, B cells, and other immune cells, amplifying synovial inflammation [252]. Early control of inflammation is crucial to prevent irreversible damage to the joints. However, conventional anti-inflammatory therapies are often associated with marked adverse effects, including an increased risk and systemic immunosuppression [253]. Therefore, an urgent need exists to develop safer, more selective, and effective treatment strategies for RA.

Given this therapeutic gap, PRLMs have garnered increasing interest as potential candidates owing to their ability to regulate inflammation and promote tissue repair. Impaired inflammatory resolution is recognized as a key pathogenic mechanism of RA. Metabolipidomic profiling using LC–MS has demonstrated that circulating levels of several PRLMs, including RvD3, RvD4, RvE3, and 15R-LXA4, are markedly reduced in patients with RA compared with those in healthy controls, with a prominent decrease in RvD3 [254]. Similarly, lower serum concentrations of RvD1, together with increased connective tissue growth factor levels, have been correlated with RA onset and progression [47]. Disease-activity–based stratification further supports these associations: individuals with inactive RA show higher circulating levels of PDX and MaR1 compared with those of healthy participants, whereas those with active disease consistently present markedly reduced levels [48]. Collectively, these findings suggest that insufficient endogenous PRLM biosynthesis contributes to RA pathogenesis and disease progression. They also indicate that peripheral PRLM signatures may serve as clinically relevant biomarkers for assessing disease activity and guiding patient stratification.

Correspondingly, the therapeutic potential of exogenous PRLMs in RA has received increasing attention. In 2016, Norling et al. [255] demonstrated that RvD1 reduces joint swelling, leukocyte infiltration, and proinflammatory lipid mediator production while increasing endogenous PRLM levels and accelerating inflammation resolution. Subsequent studies have shown that additional PRLMs, including RvD3, RvD5, PDX, MaR1, and MCTR3, produce robust anti-arthritic effects in experimental models [256]. Mechanistic evidence indicates that these mediators modulate several immune cell subsets that are central to RA pathogenesis. For instance, MCTR3 reprograms monocytes by upregulating Arg-1 expression, enabling durable pro-resolving activity and joint protection [257]. Likewise, RvD1, RvD2, PDX, and MaR1 inhibit Th17 differentiation while promoting Treg expansion, restoring the Th17/Treg balance and reducing disease severity [48]. RvD1 and RvE1 also suppress osteoclast differentiation, limiting bone resorption and preserving joint structural integrity during disease progression [258].

Besides immunomodulation, PRLMs contribute to symptom alleviation, particularly pain relief. In arthritis models with diminished endogenous MaR1 expression, exogenous MaR1 administration prevents macrophage recruitment to the dorsal root ganglia and reverses pain hypersensitivity [259]. Together, these findings suggest that endogenous PRLM levels are closely linked to disease severity and that declining concentrations may contribute to the transition from inactive to active RA. However, the relationship between circulating PRLMs and clinical progression remains unclear. Further research, particularly studies assessing PRLM concentrations in synovial fluid and correlating them with local inflammation and joint destruction, is needed to better establish their mechanistic and translational relevance.

#### Multiple sclerosis (MS)

MS is an autoimmune disorder of the CNS, characterized by inflammatory injury to the myelin sheaths and axons [260]. A hallmark of disease pathology is the extensive infiltration of inflammatory cells into demyelinating lesions, a process that is well replicated in the experimental autoimmune experimental autoimmune encephalomyelitis (EAE) model. Autoreactive T cells initiate lesion formation by releasing cytokines and chemokines that recruit and activate additional immune cell populations, ultimately driving CNS inflammation and demyelination [261]. Given this immune-driven pathology, the suppression of both local and systemic inflammation remains central to disease management. However, long-term therapeutic options are limited, and predicting patient outcomes—particularly following irreversible CNS injury—remains challenging [260]. Accordingly, identifying therapeutic strategies that can complement or improve current interventions has become a major research priority.

The concept of inflammation resolution presents substantial therapeutic promise for autoimmune disorders. However, research applying this framework to MS is relatively recent, emerging around 2013, and findings to date remain inconsistent [98]. Several studies have reported markedly reduced CSF concentrations of RvD1 in both patients with MS and EAE models, indicating deficient resolution pathways [262]. This decrease correlates with Expanded Disability Status Scale (EDSS) scores, suggesting that impaired resolution mechanisms contribute to disease pathogenesis [263]. Conversely, other studies have identified elevated circulating levels of several PRLMs, including LXA4, LXB4, AT-LXA4, RvD1, RvD5, PD1, and PDX, in patients with MS, with further increases observed in the progressive stages [262]. These mediators also showed positive correlations with EDSS scores [262]. Temporal profiling further indicated that RvD1, RvD5, and NPD1 levels increase during active disease phases but decline as inflammation transitions into chronicity [264]. Taken together, these divergent patterns likely reflect the heterogeneity in disease subtype, stage, and analytical methodology. Therefore, well-controlled longitudinal and comparative studies are essential to clarify PRLM dynamics and establish their mechanistic and clinical relevance.

Targeted lipidomic analysis of peripheral blood from patients with MS by Kooij et al. revealed that most PRLMs are markedly reduced or even undetectable as the disease advances [262]. By contrast, LXA4 and lipoxin B4 (LXB4) demonstrated protective effects by suppressing the activation and cytokine release from MS-derived monocytes in vivo, improving BBB integrity, and limiting monocyte migration [265]. Supporting these clinical findings, animal studies have shown that intraperitoneal administration of LXA4 alleviates symptoms in EAE mice, reduces CD4 + and CD8 + T-cell infiltration into the CNS, and downregulates pathogenic effector subsets, such as Th1 and Th17 cells. Notably, IL-17 secreted by Th17 cells exerts high pathogenic potential, as it disrupts the BBB and recruits additional immune cells into the CNS, amplifying inflammatory injury [266].

Besides LXA4, other PRLMs also exhibit substantial immunoregulatory potential. Exogenous MaR1 administration expands Tregs, reduces Th1 cell populations, and drives macrophage polarization toward an anti-inflammatory phenotype [267], reshaping the immune microenvironment at multiple levels. Under physiological conditions, PRLMs rise rapidly during the peak of acute inflammation, activating endogenous resolution programs that limit excessive immune responses and restore tissue homeostasis [268]. However, during acute MS relapses, excessive release of proinflammatory mediators may impair PRLM synthesis and function, resulting in defective resolution. Consistent with this, clinical studies have reported that several key PRLMs are markedly reduced or absent in patients with MS [262]. Such defects in endogenous resolution mechanisms may underlie chronic disease and contribute to irreversible neurodegeneration.

Studies investigating the role of PRLMs in MS have used both in vivo and in vitro models. In vivo, treatment with RvD1, LXA4, or MaR1 improves clinical outcomes in EAE by reducing spinal cord demyelination and preventing neuronal loss [267]. Mechanistically, PRLMs reshape the inflammatory milieu by suppressing T-cell activation and infiltration in both the peripheral and central immune compartments while promoting anti-inflammatory phenotypes in macrophages and resident microglia [267]. RvD1 administration also increases spinal myelin protein and neurotrophic factor expression, suggesting partial restoration of myelin integrity and neuronal function [263]. Complementary in vitro studies have shown that LXA4, LXB4, RvD1, and PD1 suppress monocyte activation and reduce proinflammatory cytokine production [262]. These effects were more pronounced in monocytes from healthy individuals than in those derived from patients with MS, suggesting a hyporesponsive or desensitized immune phenotype, although the mechanism remains unresolved. PRLMs additionally support BBB integrity by suppressing adhesion molecules and chemokine expression, reducing inflammation-induced barrier disruption and monocyte trafficking [262]. Collectively, these findings position PRLMs as promising modulators of neuroinflammation and immune dysregulation in MS.

MS is a classic example of pathological convergence between autoimmune and NDDs. Although the disease begins with autoimmune attacks on the myelin sheath of the CNS, the chronic inflammatory environment directly leads to progressive neurodegeneration, including axonal disruption, neuronal loss, and brain atrophy. This neurodegenerative component is the primary cause of irreversible functional impairment in advanced disease stages. Therefore, MS inherently possesses a dual nature, necessitating treatment strategies that simultaneously address both inflammatory and degenerative processes.

Deficient resolution of inflammation likely contributes to disease progression in patients with MS. Although research on PRLM biology in MS has expanded, current findings remain inconsistent, underscoring the need for continued investigation. Future research should establish the clinical relevance of PRLMs, including their association with MRI-enhancing lesions, brain atrophy, and cognitive decline. Moreover, several newly identified PRLMs have yet to be investigated, and defining their roles in MS and EAE models will be an important next step. Finally, examining how PRLMs influence remyelination and neuronal injury and extending research beyond immune cells to include astrocytes, oligodendrocytes, and neurons may provide deeper insights into their broader functions within the CNS.

#### Inflammatory bowel disease (IBD)

IBD, encompassing Crohn’s disease and UC, is a chronic immune-mediated disorder of the gastrointestinal tract that remains without a definitive cure [269]. Loss of the tightly regulated immune–epithelial equilibrium in the intestinal mucosa leads to persistent inflammation, characterized by mucosal injury, increased epithelial permeability, bacterial translocation, leukocyte infiltration, and dysregulated cytokine production [50]. Existing therapeutic options are frequently immunosuppressive, costly, and ineffective in a substantial proportion of patients [50]. With the global prevalence of IBD continuing to rise and limitations of current therapies becoming increasingly evident, innovative treatment strategies that restore tissue homeostasis by resolving inflammation are urgently required.

Tissue biopsy analyses of patients with IBD revealed elevated levels of several PRLMs, including RvD5, PD1 derived from n-3 DPA, and 10S,17S-diHDPA, compared with those in healthy controls, and these mediators demonstrated marked protective effects in experimental IBD models. Despite this elevation, endogenous PRLM biosynthesis appears insufficient, supporting the view that increasing PRLM concentrations may not be adequate to counteract the excessive inflammatory response [50]. Additional PRLMs, such as RvE1 [270], AT-RvD1 [271], and MaR1 [272], have also shown strong therapeutic efficacy in preclinical IBD settings.

Mechanistic studies suggest that PRLMs exert their protective effects by limiting leukocyte infiltration, reducing proinflammatory cytokine production, and mitigating oxidative stress–induced damage within the gastrointestinal tract, collectively reducing disease severity and colonic injury [270–272]. Reduced leukocyte trafficking is associated with the downregulation of chemokines and adhesion molecules; for instance, RvE1 suppresses MIP-2 and CXCL1 expression in colonic lesions [270], whereas AT-RvD1, RvD2, and 17R-HDHA downregulate VCAM-1 and ICAM-1 transcription [271]. PRLM treatment also decreases the secretion of inflammatory cytokines, including TNF-α, IL-1β, and IL-6, and prevents tissue injury by inhibiting inducible nitric oxide synthase, COX-2, and myeloperoxidase activation [270–272]. These effects are thought to involve the negative regulation of NF-κB signaling [270], although further mechanistic clarification is needed.

Together, the current findings indicate that PRLM insufficiency contributes to ongoing inflammation in IBD and highlights their considerable disease-modifying potential. However, most research has primarily focused on their anti-inflammatory roles. Future studies investigating PRLM involvement in tissue repair and mucosal regeneration are essential to define their therapeutic relevance.

#### Psoriasis (PSO)

PSO is a common immune-mediated skin disease characterized by excessive keratinocyte proliferation and prominent inflammatory cell infiltration [273]. Activation of monocytes, DCs, and macrophages drives the release of key cytokines, including IL-1, IL-6, TNF-α, IL-17, and IL-23, which together establish a core inflammatory network that sustains disease development and progression [274]. Within this network, the IL-23/IL-17 axis has emerged as a central regulator, critically contributing to both the initiation and chronic maintenance of PSO [275]. Although current therapies have improved clinical outcomes, the pathogenic mechanisms remain incompletely understood, and treatment resistance or relapse is still frequently observed. Refining mechanistic understanding and developing more precise therapeutic strategies remain important research priorities in this field.

In psoriatic skin, an imbalance between pro-resolving and proinflammatory lipid mediators indicates their contribution to disease pathogenesis. Lesional tissue demonstrates elevated levels of RvD5, PDX, AT-LXA4, and AT-LXB4, whereas only PD1 is detectable in healthy skin [276], suggesting altered lipid mediator signaling in PSO. Functional evidence further supports this shift: RvD1 and RvD5 suppress proinflammatory cytokines, including IL-1β, TNF-α, IL-24, and S100A12, in human keratinocytes, reinforcing the therapeutic relevance of PRLMs [276].

Mechanistically, PRLMs reduce immune cell infiltration into psoriatic lesions, limit inflammatory and lymphocyte migration, and suppress cytokine production, collectively contributing to improvements in clinical symptoms, such as skin thickening, erythema, and scaling. A key element of their therapeutic effect is the modulation of the IL-23/IL-17 axis, which is a fundamental pathway in PSO pathophysiology [277]. Although MaR1 does not directly inhibit IL-23, it decreases IL-23 receptor expression on T cells, dampening downstream signaling [278]. Complementing this immunomodulatory role, RvD3 alleviates TRPV1-dependent pain and spontaneous itch by reducing calcitonin gene-related peptide expression in dorsal root ganglia neurons [279]. Together, these findings indicate that PRLMs not only regulate central inflammatory mechanisms but also relieve inflammation-associated discomfort, supporting their continued investigation as a promising therapeutic approach.

#### Type 1 diabetes mellitus (T1DM)

T1DM is an autoimmune disease that accounts for approximately 10% of all diabetes cases [280]. The disease is characterized by the infiltration of monocytes, macrophages, and lymphocytes into pancreatic tissue, leading to the release of proinflammatory cytokines, such as IL-1, IL-2, IL-6, and TNF-α. These inflammatory mediators induce β-cell apoptosis, impair insulin secretion, and ultimately result in hyperglycemia [281]. Thus, controlling inflammation is a critical therapeutic strategy for preventing β-cell loss and improving clinical outcomes and long-term prognosis in patients with T1DM.

Besides immunological mechanisms, epidemiological evidence highlights potential preventive dietary factors. Large population-based case–control studies have indicated that cod liver oil supplementation during pregnancy or infancy is associated with a reduced risk of childhood T1DM [282]. Similarly, prolonged breastfeeding markedly decreases T1DM susceptibility. As both cod liver oil and breast milk contain high levels of PUFAs [283], these observations suggest a protective role of PUFAs in modifying disease risk. Further support comes from experimental studies: fat-1 transgenic mice, which endogenously synthesize n-3 PUFAs, exhibit marked resistance to T1DM compared with wild-type controls [284]. This resistance correlates with elevated pancreatic levels of anti-inflammatory mediators, including LXA4 and 18-hydroxyeicosapentaenoic acid [284]. Together, these findings indicate that PUFAs, particularly their downstream PRLMs, may serve as promising therapeutic or preventive candidates for T1DM.

Building on this concept, interest has increasingly focused on the therapeutic relevance of PRLMs in T1DM. PRLMs facilitate inflammation resolution and tissue repair without compromising host immune defense, making them attractive modulators of disease. Evidence from experimental T1DM models has demonstrated that LXA4 and RvD1 markedly reduce disease severity [285]. Their protective effects involve multiple mechanisms, including the restoration of inflammatory gene regulation (such as NF-κB signaling), enhancement of antioxidant defense systems (e.g., superoxide dismutase 1, catalase, glutathione S-transferase, and glutathione peroxidase), and normalization of lipid peroxidation and nitric oxide (NO) levels [285]. Moreover, both mediators increase the expression of pancreatic and duodenal homeobox-1 (PDX-1), a transcription factor essential for pancreatic β-cell development and maturation [285]**.** Collectively, these findings suggest that PRLMs may delay or attenuate T1DM progression by reducing inflammation and oxidative stress while promoting β-cell regeneration.

#### Sjögren’s syndrome (SS)

SS is a common autoimmune disorder characterized by chronic inflammation and progressive destruction of exocrine glands, leading to symptoms such as xerostomia and keratoconjunctivitis sicca [286]. Although the precise mechanisms underlying glandular injury in primary SS remain unclear, current evidence suggests that disease onset and progression may follow a multistep pathogenic process similar to that of other autoimmune conditions [286]. Available therapeutic options are limited, and numerous patients show suboptimal responses to supportive treatment or systemic immunosuppression, resulting in inadequate disease control. Therefore, a clear unmet need exists for the development of more effective and targeted therapeutic strategies against SS.

In this clinical context, recent mechanistic studies have revealed distinct lipid mediator dynamics in SS. In contrast to many other autoimmune diseases, plasma and saliva from female SS animal models collected after disease onset contain markedly elevated levels of several PRLMs, particularly RvD1 and RvD2, along with increased expression of their biosynthetic enzymes and corresponding receptors [287]. Consistent with these observations, RvD1, AT-RvD1, and LXA4 have been shown to facilitate inflammation resolution and promote tissue repair in both in vitro and in vivo SS models [288]. Mechanistically, these mediators suppress proinflammatory cytokine production, reduce the recruitment and infiltration of Th1 and Th17 cells, and increase the proportion of Tregs [288]. They further inhibit epithelial cell apoptosis, enhance cell migration and polarity, and support tissue regeneration [289]. In addition, they can reverse inflammation-induced disruption of tight junctions and cytoskeletal organization [289].

Taken together, these findings indicate that although PRLM levels are elevated in SS-like mouse models, likely as a transient compensatory response to excessive inflammation, this endogenous increase is insufficient to resolve the inflammatory process. The substantial therapeutic effects observed following exogenous PRLM administration further reinforce their potential as promising treatment candidates for SS.

#### Systemic lupus erythematosus (SLE)

SLE is a chronic autoimmune disease with an unclear etiology that is potentially linked to persistent and unresolved inflammation [290]. Environmental and genetic factors are believed to drive abnormal immune activation, resulting in disrupted T-cell homeostasis, excessive production of pathogenic autoantibodies by B cells, and dysregulated cytokine signaling, ultimately causing tissue and organ damage [290]. Increasing evidence suggests that the resolution phase of inflammation is markedly impaired in patients with SLE. For example, plasma levels of RvD1 are markedly reduced in patients compared with those in healthy controls and show a negative correlation with disease severity, with further decreases observed during active disease [93]. Functionally, RvD1 suppresses Th17 differentiation and promotes the conversion of naïve CD4⁺ T cells into Tregs, restoring the Treg/Th17 balance. In lupus-prone MRL/lpr mice, RvD1 treatment lowered serum anti-dsDNA IgG levels, indicating its potential capacity to limit pathogenic autoantibody production [93].

Building on these observations, additional mechanistic studies have further supported the regulatory role of PRLMs in SLE. Although PRLMs enhance protective antibody responses under physiological conditions, they suppress autoreactive antibody production in autoimmune contexts, suggesting that B cells may exist in distinct functional states in health and disease—an area requiring further investigation. In addition, RvD1 treatment increases the levels of the anti-inflammatory cytokine IL-10 while reducing proinflammatory mediators such as TNF-α, IFN-γ, IL-6, and IL-17, reflecting suppressed inflammatory activity and reduced tissue injury [93]. Together, these findings support the concept that defective inflammatory resolution contributes to SLE pathogenesis and that exogenous PRLM supplementation may offer therapeutic benefits.

PRLM-based therapies hold promise for modulating endogenous repair pathways while minimizing adverse effects, as they do not interfere with natural host immunity. However, further studies are required to clarify the roles of individual mediators across distinct immune cell subsets, as specific PRLMs may activate different pro-resolution pathways and induce heterogeneous immune responses during inflammation. Defining their functions in mature and differentiated immune cells is important for understanding how PRLMs regulate inflammation resolution. Furthermore, evaluating more stable and selective PRLM analogs may help determine their therapeutic potential in restoring impaired resolution processes associated with diverse human diseases. Advances in detection technologies will also facilitate the mapping of disease-specific PRLM expression patterns, and continued research into their immunoregulatory functions will support the development and refinement of clinical applications for autoimmune and inflammatory disorders.

## Clinical translation potential and optimization of therapeutic strategies for PRLMs

**Fig. 6:**
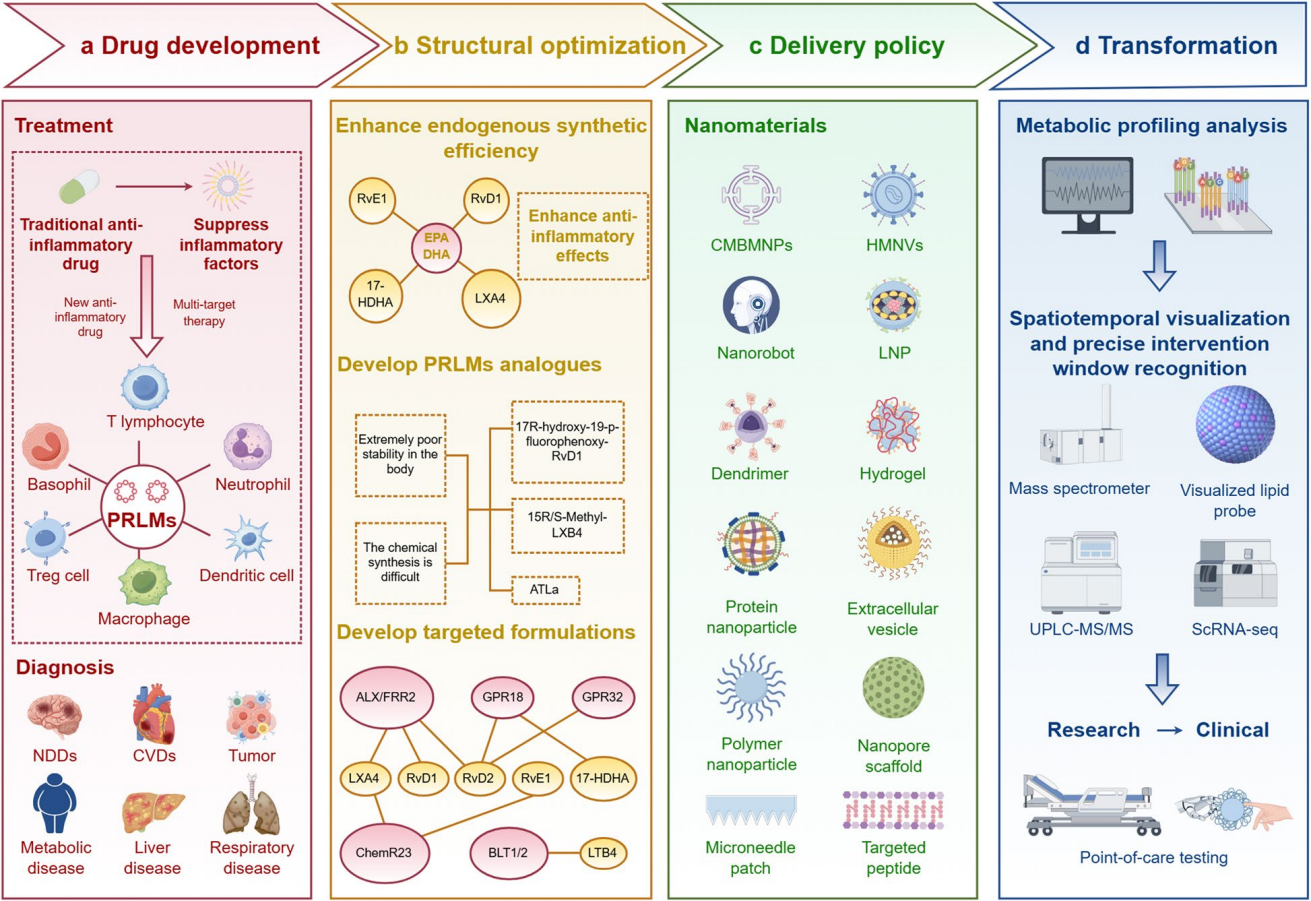
Future perspectives and translational strategies for PRLMs (By Figdraw, License ID: UTIRT1b244). The figure summarizes key directions for advancing PRLMs-based research and clinical translation. **a** Drug development: PRLMs are envisioned to complement or replace conventional anti-inflammatory therapies by actively regulating immune responses through multi-target modulation of immune cells across cardiovascular, neurodegenerative, metabolic, hepatic, and respiratory diseases. **b** Structural optimization: Current efforts focus on enhancing endogenous biosynthesis, improving molecular stability, and developing receptor-selective analogues such as 17R-RvD1, 15R/S-methyl-LXA4, and ATLa. **c** Delivery policy: Innovative nanomaterial platforms—including biomimetic nanoparticles, dendrimers, hydrogels, and extracellular vesicles—enable targeted and sustained delivery in vivo. **d** Clinical transformation: Integration of metabolic profiling, lipidomics, and single-cell sequencing facilitates spatiotemporal visualization of inflammation and promotes precision interventions bridging basic and clinical research. Abbreviations: PRLMs, pro-resolving lipid mediators; RvD1, Resolvin D1; RvE1, Resolvin E1; LXA4, Lipoxin A4; ATLa, Aspirin-triggered lipoxin A4; 17-HDHA, 17-hydroxy-docosahexaenoic acid; ALX/FPR2, Annexin A1 receptor/Formyl peptide receptor 2; ChemR23, Chemerin receptor 23; GPR18, G-protein-coupled receptor 18; GPR32, G-protein-coupled receptor 32; BLT1/2, Leukotriene B4 receptor 1/2; CMBMNPs, cell membrane biomimetic nanoparticles; HMNVs, human microvesicle nanovesicles; LNP, lipid nanoparticle; EVs, extracellular vesicles; UPLC-MS/MS, ultra-performance liquid chromatography–tandem mass spectrometry; scRNA-seq, single-cell RNA sequencing

Although PRLMs have demonstrated considerable therapeutic promise in modulating inflammation and promoting tissue repair across diverse disease models, their translation into clinically viable drugs remains challenging [4]. Endogenous PRLMs are structurally unstable, rapidly metabolized, and possess short half-lives in vivo, limiting their direct application as therapeutic agents. In addition, the complex synthesis and low yield of natural PRLMs pose obstacles to large-scale development. To overcome these limitations, research has increasingly focused on structural optimization and synthetic strategies, leading to the generation of PRLM analogs and receptor agonists with improved pharmacokinetic profiles and therapeutic efficacy. These advances provide an essential foundation for clinical translation (Fig. 6). Table 2 summarizes recent clinical studies investigating the therapeutic potential of PRLMs in inflammation resolution.

**Table 2:** Representative clinical studies currently conducted on strategies targeting chronic inflammation

Agents	Purpose/Result	Status	Register ID
ω−3 fatty acids	Evaluating the impact of ω−3 fatty acids on the production of anti-inflammatory effects and clinical improvement in people with depression who have not responded well to standard antidepressant treatment	Recruiting	NCT05774665
ω−3 DHA	Assessing the effectiveness of providing additional DHA ω−3 fatty acids supplements to increase the levels of pro-resolving	Completed	NCT01865448
Montelukast	Evaluating SPM on lipid profiles and TAMs in patients with cancer	Not recruiting	NCT06887673
Lovaza	Effect of ω−3 fatty acids on the formation and function of PRLMs in acute inflammation	Completed	NCT04308889
SPM Active®	Learning if daily supplementation with SPM Active® can increase ω−3 PUFA derivatives and improve well-being in adults with obesity	Recruiting	NCT06991296
ω−3 fatty acids	Effects of high-dose ω−3 fatty acid supplementation on serum apolipoprotein A levels and its association with muscle function and muscle injury markers after high-intensity eccentric exercise	Completed	NCT07237113
N-of-1 Tent-Umbrella-Bucket	To evaluate the impact of personalized lifestyle interventions on functional capacity, and its relationship with known disease risk determinants	Completed	NCT04005456
ω−3 fatty acids	To evaluate its in vitro/in vivo regulatory effects, the mechanisms of chronic regulation, and its role in promoting anti-inflammatory and neuroprotective pathway activation	Completed	NCT03492606
SPM Active	Comparison of plasma SPM concentration and immune adaptability in patients with obesity after oral and oral injection	Completed	NCT04701138
SPM	To investigate whether there are genetic variations (mutations) in the genes encoding the enzymes and receptors and to determine how these mutations affect the activity or function of SPM	Recruiting	NCT04698291
Aspirin	The effects of body weight and aspirin dose on the levels of specific proinflammatory mediators in blood were discussed	Completed	NCT04697719
SPM Emulsion	Effects of fish oil supplements on inflammation	Completed	NCT02719665
LIPINOVA	Evaluating the comparison of SPM in assessing pain, stiffness, and functional changes in osteoarthritis	Completed	NCT05633849

Data sources: clinical registration website (https://clinicaltrials.gov)

*Abbreviations*: *SPM* Specialized pro-resolving mediator, *DHA* Docosahexaenoic acid, *TAMs* Tumor-associated macrophages, *ω−3* Omega-3, *PUFA* Polyunsaturated fatty acid

### PRLMs as diagnostic and prognostic biomarkers

PRLMs are endogenous lipid mediators with multi-target immunoregulatory functions that have advanced from basic research to preclinical evaluation and the early stages of drug development. Unlike traditional anti-inflammatory agents, which primarily suppress inflammatory responses, PRLMs regulate resolution pathways, enhance macrophage-mediated clearance, and promote tissue regeneration. Collectively, these mechanisms support a therapeutic strategy aimed at the “reconstruction of immune homeostasis” [291]. This shift from an “anti-inflammation” to “pro-resolution” paradigm highlights the potential of PRLMs as promising candidates for managing complex conditions, such as chronic inflammation, immune dysregulation, and multi-organ injury.

Further mechanistic studies indicate that PRLMs are not merely anti-inflammatory molecules. Unno et al. [292] demonstrated that RvE1 binds to LTB4 receptors on neutrophils, moderately stimulating ROS production and thus exerting limited proinflammatory activity. Unlike classical mediators such as TNF-α, the activity of RvE1 is tightly confined within an “immune-moderate response” range, preventing excessive inflammatory damage. This bidirectional mechanism, defined as “mild proinflammation followed by rapid resolution,” underscores the distinctive advantages of PRLMs in balancing immune responses in complex environments.

Besides mechanistic regulation, PRLMs have attracted interest as clinical biomarkers. In a prospective cohort study of 59 critically ill children admitted to the ICU, netrin-1 promoted the synthesis of PRLMs, including LXA4, MaR1, and PDX. Plasma netrin-1 concentrations were inversely correlated with ICU length of stay and mortality, suggesting its potential as a predictor of disease severity and prognosis in critical illness [293]. Extending these findings, Dalli et al. [294] profiled PRLMs dynamically in 22 patients with sepsis, showing that plasma levels of RvE1, RvD5, and 17R-PD1 were markedly higher in no survivors than in survivors. Moreover, in patients with ARDS, PDX levels on day 3 outperformed the APACHE II score in predicting disease progression. Together, these results highlight PRLMs as functional molecular biomarkers for evaluating sepsis and its complications.

At the therapeutic level, PRLMs also demonstrate the potential for synergistic effects with existing drugs. In a study of 50 patients with diabetic chronic kidney disease, Goicoechea et al. [295] administered low-dose aspirin to evaluate changes in 15-epi-LXA4 (aspirin-triggered LX, ATL). Baseline ATL concentrations were markedly lower in patients with diabetes than in nondiabetic controls; however, 12 months of low-dose aspirin therapy markedly increased ATL levels. These findings suggest that PRLMs may function as modulators of immune reprogramming induced by established agents such as aspirin, enhancing their anti-inflammatory efficacy.

Although PRLMs show considerable promise for treating severe infections, autoimmune disorders, and chronic inflammatory diseases, direct clinical evidence for their therapeutic application remains limited. Future research should focus on improving their stability, delivery systems, and target specificity, as well as conducting large-scale, multicenter clinical trials. PRLMs are emerging as central components of next-generation immunoregulatory strategies that integrate diagnosis, therapy, and prognosis, and their translational potential warrants comprehensive exploration.

### Therapeutic strategies and drug development

The clinical translation of PRLMs is constrained by their intrinsic biological properties, which pose marked challenges. Endogenous PRLMs are metabolically unstable, exhibit short half-lives, are difficult to synthesize, and have low delivery efficiency. These limitations severely restrict their therapeutic application. To address these barriers, researchers have proposed a range of optimization strategies, including enhancing endogenous biosynthesis, developing structurally stable analogs, designing receptor-targeted modulators, and novel delivery systems and personalized therapies. Together, these approaches have advanced PRLM research from fundamental studies to clinical interventions [296]. The following subsections outline these strategies, providing both theoretical foundations and technical pathways for drug development and translational medicine.

#### Enhancing endogenous synthesis efficiency

Activating endogenous PRLM biosynthetic pathways increases local concentrations and reinforces physiological functions. Supplementation with ω−3 PUFAs enriched in DHA and EPA has been shown to promote the generation of PRLMs, including RvE1, RvD1, LXB, 18-HEPE, and 17-HDHA, enhancing anti-inflammatory and tissue-repair activity [83]. In healthy volunteers, LPS stimulation typically induces an early decline in PRLM levels, which recovers within 24 h. By contrast, in individuals receiving ω−3 supplementation, PRLMs rose rapidly and peaked within 2 h of stimulation, suggesting that substrate availability accelerates early PRLM production. Mechanistic insights further support this hypothesis. In human polymorphonuclear cells, Unno et al. [292] demonstrated that RvE1 moderately activated neutrophil oxidative function through LTB4 receptors while preventing excessive inflammation, reflecting a “mild proinflammatory” profile that helps maintain immune responses within a controlled range. Clinical evidence has also emerged: in a cohort of critically ill children, plasma levels of neuronal guidance protein correlated positively with the production of LXA4, MaR1, and PDX, whereas being inversely associated with ICU length of stay and mortality [293]. Collectively, these findings suggest that PRLMs not only reinforce resolution pathways but may also serve as predictive biomarkers of disease severity and clinical outcomes.

#### Development of structurally stable PRLM analogs

Despite the promise of enhancing endogenous synthesis, the therapeutic use of natural PRLMs is still limited by their rapid metabolic degradation and synthetic complexity, underscoring the need for structurally stable analogs. LXA4 mimetics, such as 15R/S-methyl-LXB4 and ATLa, incorporate methylation and aromatic modifications to resist metabolic inactivation and have demonstrated therapeutic efficacy in chronic inflammatory models, including those of eczema and asthma. Metabolically stable RvD1 analogs, such as 17R-hydroxy-19-p-fluorophenoxy-RvD1, have also shown protective effects in lung injury models by modulating the local inflammatory microenvironment. Other structural modifications, including the replacement of the triene core with a benzene ring, have yielded promising results in models of diabetic nephropathy and AS [295]. Clinical observations further support this hypothesis. In patients with diabetic chronic kidney disease, low-dose aspirin induces the generation of 15-epi-LXA4 and elevated levels correlate with clinical benefits. These examples highlight structural optimization as a pivotal strategy for enhancing the stability, efficacy, and translational potential of PRLM-based therapies [295]. Extending from these conceptual and mechanistic insights, PRLM analogs, such as RvE1 mimetics, have advanced to phase I/II clinical trials, primarily to assess long-term safety, pharmacokinetics, and therapeutic windows [18]. In a randomized controlled trial involving healthy volunteers, researchers induced an acute inflammatory response with a low dose of lipopolysaccharide (LPS) and monitored changes in the lipid mediator profile under conditions of ω−3 PUFA supplementation. LPS stimulation markedly increased proinflammatory lipids, including prostaglandins and thromboxanes, whereas PRLMs showed a transient decline during the early phase of inflammation (within 2 h), followed by a marked increase at 24 h. By contrast, in participants receiving ω−3 supplementation, PRLMs such as RvE1, RvD1, LXB, 18-HEPE, and 17-HDHA increased rapidly and peaked within 2 h of the LPS challenge [83]. These findings suggest that the transient downregulation of PRLMs may be required to initiate inflammation, whereas ω−3 fatty acids enhance early PRLMs induction, providing insight into the mechanisms of immune homeostasis.

#### Development of small-molecule agonists and antagonists targeting PRLM receptors

Besides the chemical modification of PRLMs, targeting their receptors directly represents another promising strategy. PRLMs primarily mediate their biological actions through GPCRs, including ALX/FPR2, GPR18, and GPR32. Receptor expression is often downregulated in disease states, suggesting that receptor agonists or antagonists could restore or enhance PRLM-mediated effects. FPR2 agonists, such as compound 17b, BMS-986235, and WKYMVm, have demonstrated reparative activity and reduced immune dysregulation in experimental models of myocardial infarction (MI) and HF [297–300]. GPR18 agonists, including PSB-KD-107, enhance NO production and alleviate vasoconstriction, conferring vascular protection in models of pulmonary and systemic hypertension [301]. Similarly, GPR32 agonists, such as AT-RvD1, promote macrophage polarization and lipid clearance in AS. Antagonists also hold therapeutic potential; ChemR23 inhibitors (e.g., CCX832) and LTB4 receptor blockers (e.g., CP-105,696 and LY293111) have demonstrated efficacy in models of diabetic vascular complications and IRI [302, 303]. Together, these findings underscore receptor-targeted modulation as an effective strategy for expanding the therapeutic scope of PRLM-based interventions.

### Novel delivery systems and personalized therapies

The primary goal of PRLM-based strategies for resolving inflammation is to increase their concentrations at sites of injury rather than throughout the systemic circulation. Achieving this requires the development of targeted delivery systems capable of directing therapeutics specifically to damaged tissues, reducing systemic toxicity and enhancing efficacy. Nevertheless, small-molecule compounds targeting PRLM receptors still face major challenges, including unfavorable pharmacokinetics and insufficient cardiac specificity, both of which may contribute to adverse effects.

#### Nanoparticle-based delivery systems

The therapeutic application of PRLMs in chronic inflammatory diseases is limited by their restricted tissue-targeting capacity. To overcome this barrier, researchers have developed nanoparticle-based platforms, including LNPs, cell membrane biomimetic nanoparticles (CMBMNPs), and exosomes, to transport PRLMs or their agonists directly to injured tissues. These systems have yielded promising results in several disease models. For instance, encapsulating RvD1 in liposomes markedly prolonged its half-life and improved its stability, whereas exosome-mediated delivery of Rvs accelerated wound healing in diabetic ulcer models [79]. Despite these advances, conventional liposomes and native exosomes still face major limitations, including restricted drug-loading capacity, rapid clearance by the mononuclear phagocyte system, and limited targeting efficiency [304, 305].

To address these limitations, multifunctional hybrid membrane nanovesicles (HMNVs) have become a major focus. HMNVs derived from neutrophil membranes can deliver RvD1, AT-RvD1, RvD2, and LXA4 mimetics to sites of inflammation with high specificity, modulating the local inflammatory microenvironment. To further enhance stability and targeting, researchers have investigated exosome surface modifications with homing peptides, such as the cardiac-homing peptide (CHP), or the incorporation of immune-evasion signals, such as CD47, which reduces rapid clearance in vivo [306]. In parallel, biomimetic liposomal systems have demonstrated strong tissue-targeting ability, particularly in cardiac disease models, where they achieve local accumulation and exert anti-inflammatory effects. More advanced technologies, including nanorobots, are emerging in precision medicine [262]. These systems offer autonomous navigation, the capacity to cross biological barriers, and the ability to release drugs directly at the sites of injury, presenting promising opportunities for intelligent PRLM delivery.

#### Hydrogel-based drug delivery platform

Hydrogels, as three-dimensional network scaffolds, have attracted considerable interest for localized PRLM delivery owing to their excellent biocompatibility, biodegradability, drug-loading capacity, and controlled-release properties [307]. Compared with systemic administration, hydrogel-based local therapies enable sustained drug retention and release, reducing toxicity and avoiding first-pass hepatic metabolism.

Several studies have demonstrated their therapeutic potentials. Thermosensitive isocyanide peptide hydrogels successfully encapsulated LXA4, extending its half-life. Hydrogels loaded with the RvE1 analog RX-10045 alleviated corneal inflammation [308], whereas polyethylene glycol (PEG)-based hydrogels facilitated AT-RvD1 delivery, promoting macrophage reparative polarization and wound healing [309]. Additional approaches include collagen-based 3D nanoporous scaffolds combined with Pluronic F127 for sustained RvD1 release in bone defect repair and acetylated cyclodextrin hydrogels designed to improve RvE1 delivery efficiency and local anti-inflammatory effects [310].

Given the lipophilic nature of PRLMs, cyclodextrin-based hydrogels—with hydrophobic cavities and hydrophilic shells—are promising carriers for enhancing solubility and stability [311]. Looking ahead, stimuli-responsive hydrogels capable of on-demand pulsatile PRLM release, particularly when integrated with personalized devices such as microneedles or cardiac patches, represent an innovative strategy for advancing tissue repair and cardiovascular therapy.

#### Targeted implants and personalized drug delivery models

In preclinical studies, PRLMs are most often administered via intraperitoneal or intravenous injections. Although efficient, these approaches provide limited local delivery and substantial systemic exposure, restricting their clinical applicability. To address these shortcomings, several studies have explored the incorporation of PRLMs into implantable materials for localized release. For example, AT-RvD1 has been encapsulated within electro spun PCL scaffolds to construct artificial blood vessels, which, when applied in aortic bypass surgery, reduced inflammation and promoted tissue regeneration [115]. In rabbit models, RvD1-loaded devices fabricated from poly lactic-co-glycolic acid (PLGA) films markedly inhibited neo-IH and inflammatory responses [312]. Other strategies, such as incorporating PRLMs into stents, balloons, or microcatheter systems for intramyocardial injection, have emerged as potential approaches for targeted cardiac delivery [313]. Compared with conventional implants, PRLM-modified devices not only attenuate postoperative inflammation but may also reduce implant-associated immune rejection, providing dual benefits for tissue repair and long-term functional recovery.

Taken together, advances in nanoparticle systems, hydrogel platforms, and implant-based delivery highlight complementary strategies to overcome the intrinsic limitations of PRLMs. While nanoparticles enable precise targeting and dynamic modulation, hydrogels provide sustained release and tunable responsiveness, and implantable devices ensure long-term local delivery and tissue integration. The integration of these approaches, particularly when combined with emerging precision technologies, is expected to maximize therapeutic efficacy, minimize adverse effects, and accelerate clinical translation of PRLMs. This convergence marks an important step toward the realization of personalized and precision medicine in inflammation resolution and cardiovascular therapies.

## Challenges in the clinical translation of PRLMs and future research directions

### Challenges in the clinical translation of PRLMs

As our understanding of inflammation resolution advances, the therapeutic potential of PRLMs across a wide range of diseases has become increasingly evident. Despite their promise, clinical translation remains limited by several obstacles. These include complex biosynthetic pathways, instability and heterogeneity of metabolic intermediates, and modulation of bioactivity by tissue specificity and disease stage. This structural and functional complexity hampers the precise characterization of their spatiotemporal dynamics in vivo and complicates the establishment of standardized dosing regimens and therapeutic windows.

A striking example of these challenges is provided by a recent animal study investigating the effects of RvE1 on MI. The results revealed a “double-edged sword” effect: administration of RvE1 on days 1–7 post-MI improved cardiac function, whereas the same dosage administered on days 7–14 markedly impaired recovery. These contrasting outcomes highlight the time-dependent effects of RvE1 on immune cell infiltration and function. Early intervention suppressed the infiltration of proinflammatory Ly6C^high^ monocytes/macrophages and reduced the release of inflammatory cytokines, limiting cardiomyocyte apoptosis. By contrast, late intervention likely impedes the recruitment of reparative Ly6C^low^ macrophages and expression of proangiogenic factors, ultimately suppressing neovascularization and tissue repair. This finding underscores the dynamic and time-sensitive nature of inflammation progression and resolution, emphasizing the necessity of accounting for both disease stage and immune phenotype when applying PRLMs in clinical settings [80].

**Fig. 7:**
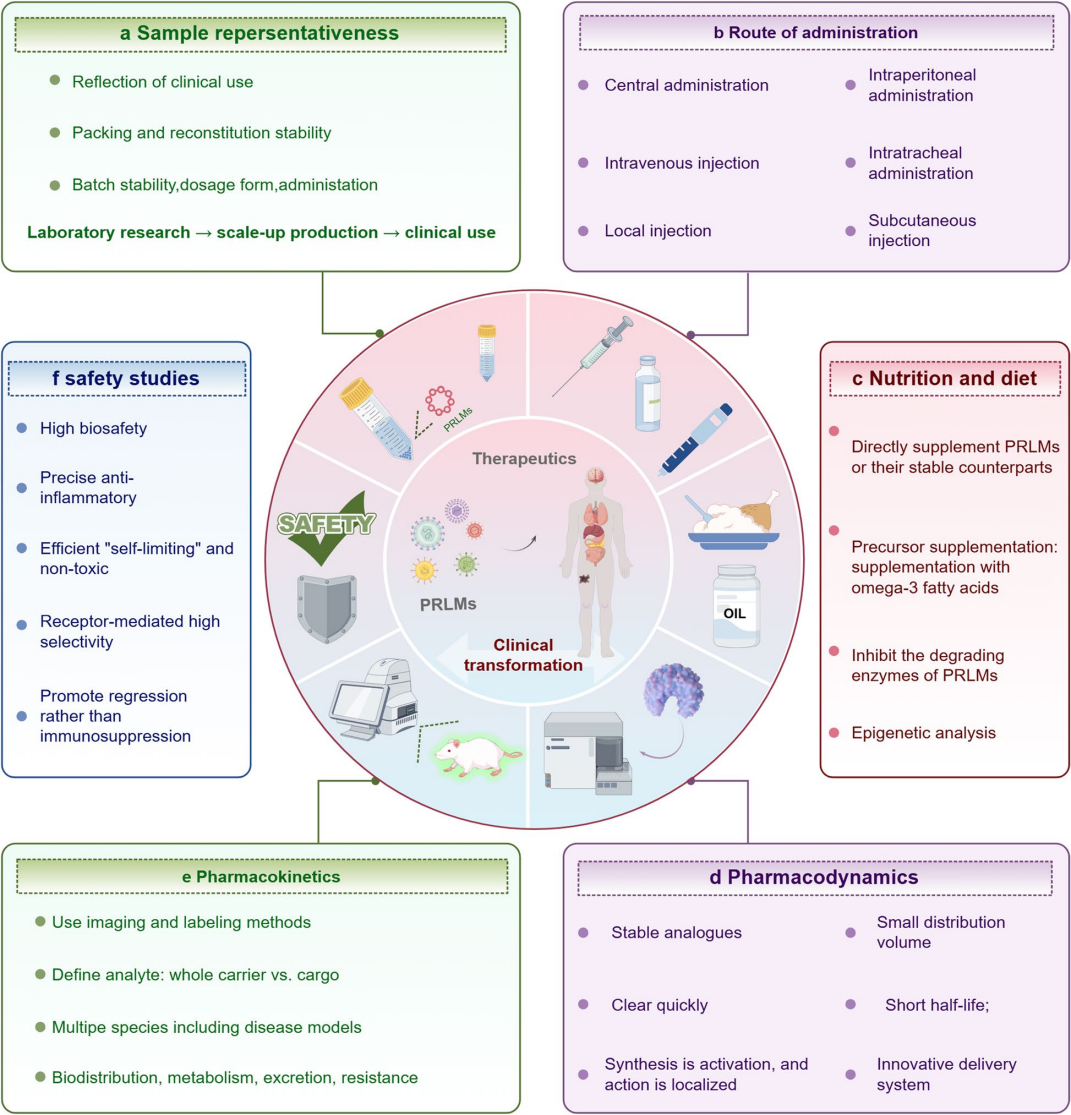
Key considerations for the clinical translation of PRLMs (By Figdraw, License ID: YPIUWa47ea). This figure summarizes major factors that influence the preclinical and translational development of PRLMs-based therapies. **a** Sample representativeness: Experimental preparations should reflect clinical conditions, with attention to stability, reproducibility, and scalable production. **b** Route of administration: Central, intravenous, intraperitoneal, intratracheal, subcutaneous, or local delivery routes determine bioavailability and tissue distribution. **c** Nutrition and diet: Clinical modulation may involve PRLMs supplementation, ω−3 fatty acid precursors, inhibition of degradation pathways, or epigenetic regulation. **d** Pharmacodynamics: PRLMs have short half-lives and limited distribution; stable analogues and optimized delivery systems can improve therapeutic efficacy. **e** Pharmacokinetics: Imaging and tracer studies help define biodistribution, metabolism, and clearance under different pathological conditions. **f** Safety assessment: PRLMs demonstrate favorable biosafety and receptor selectivity, providing anti-inflammatory effects without systemic immunosuppression. Abbreviations: PRLMs, pro-resolving lipid mediators; ω−3 FA, omega-3 fatty acids; LOX, lipoxygenase; COX, cyclooxygenase; PK/PD, pharmacokinetics/pharmacodynamics

Simultaneously, the metabolomic profiles of PRLMs and their derivatives are highly complex. Current technologies are inadequate for high throughput, dynamic, and spatially resolved quantitative analyses at the tissue level. Moreover, the lack of standardized assay kits and detection platforms restricts the use of PRLMs as therapeutic agents or biomarkers in clinical practice. To address these challenges, future research should integrate advanced methodologies and precision medicine principles within a structured framework. The following subsections outline the three key directions (Fig. 7).

### Future research directions

#### Constructing a dynamic metabolic atlas of lipid mediators–PRLMs

The synthesis, conversion, and degradation of PRLMs are tightly regulated by multiple enzymatic systems, including ALOX5, ALOX12/15, COX-2, 15-PGDH, and sEH [314]. The expression and activity of these enzymes vary across tissues and change dynamically during the progression of diseases. Constructing a comprehensive dynamic metabolic atlas of PRLMs would help identify key bottlenecks and regulatory nodes, providing a theoretical framework for targeted therapeutic development.

From a technical perspective, ultra-performance liquid chromatography–tandem mass spectrometry (UPLC-MS/MS) enables quantitative time–concentration analyses of PRLMs and their precursors or metabolites. When combined with stable isotope labeling, this approach allows the tracing of fatty acid substrates (e.g., DHA and EPA) through their conversion pathways and product lineages in disease models, facilitating the construction of individualized lipid mediator–PRLM network maps [315].

The integration of single-cell transcriptomics, enabled using single-cell RNA sequencing (scRNA-seq), with spatial transcriptomics provides high-resolution mapping of PRLM metabolism–related enzyme expression across immune cell subsets [315]. This combined approach offers valuable insights into cell type–specific biosynthetic capacities and their interactions with the inflammatory microenvironment. Collectively, this multidimensional “enzyme–product–cell–tissue” framework holds strong potential as a reference for mechanistic studies and precision therapies.

#### Spatiotemporal visualization of inflammation and identification of precise intervention windows

The functions of PRLMs strongly depend on the spatiotemporal context, often producing opposite biological effects at different stages of inflammation or within distinct tissue microenvironments. Accurately identifying the transition between the “proinflammatory” and “pro-resolving” phases is therefore critical for therapeutic design.

To achieve this, lipid-based fluorescent probes combined with imaging modalities, such as near-infrared live imaging, photoacoustic imaging, or mass spectrometry imaging, allow for the dynamic localization and quantification of PRLMs in vivo or in ex vivo tissues. These approaches can reveal the spatial distribution and activity patterns across pathological stages. In parallel, AI-driven radiomics can extract high-dimensional features related to inflammation resolution from CT, MRI, or PET scans. When integrated with PRLM levels, clinical indicators, and immune profiling data, these features can be used to construct predictive models that identify an individual’s “optimal therapeutic window” or “response subtype” [316].

In addition, dynamic algorithmic models conceptualized as “inflammatory clocks” can integrate peripheral blood PRLM profiles with immune parameters, enabling noninvasive monitoring of inflammatory cycles. Such models provide a promising basis for developing personalized and temporally precise intervention strategies.

#### Development of high throughput detection platforms and novel biomarkers

The clinical application of PRLMs is further limited by complex detection procedures, high costs, and a lack of standardization. Overcoming these barriers requires the development of detection platforms with high sensitivity, specificity, and automation, along with standardized workflows applicable to both research and clinical contexts.

Targeted lipidomics has advanced considerably in recent years. The integration of immunoaffinity enrichment with LC–MS/MS has become a widely adopted method for the quantitative analysis of PRLM family members. This approach enables highly sensitive measurements across diverse biological samples, including blood, urine, and tissue fluids, supporting large-scale cohort studies, disease subtype stratification, and early diagnosis. Beyond targeted lipidomics, emerging technologies such as lipid microarrays and electrochemical sensors provide promising opportunities for point-of-care testing (POCT). These platforms offer rapid turnaround, low sample requirements, high throughput, and multiplex detection, making them particularly useful for monitoring dynamic inflammatory states and guiding personalized therapeutic strategies [104].

Building on these advances, researchers have explored auxiliary or alternative biomarkers associated with PRLM metabolic pathways to circumvent the instability and complexity of direct PRLM measurement. For example, the expression or activity of key enzymes, such as ALOX15, COX-2, and sEH, along with upstream regulators, including non-coding RNAs, such as miR-146a, miR-21, and lncRNA MALAT1, can serve as proxies for PRLM signaling activity. These biomarkers not only act as indirect indicators of resolution potential but also provide tools to evaluate disease progression, predict therapeutic responses, and guide indication selection and target identification for PRLM-based therapies [29].

Integrating lipidomics, transcriptomics, and metabolomics with artificial intelligence and systems biology approaches enables the development of predictive models that generate a “Resolving Index.” These models can identify potential beneficiaries, refine prognostic assessments, and optimize therapeutic strategies.

Looking forward, PRLM detection platforms must also overcome practical challenges, including multicenter consistency, standardized sample processing, and harmonized data interpretation, while ensuring seamless integration into clinical workflows. Strategic collaboration with in vitro diagnostic companies to co-develop instrument platforms, reagent kits, and clinical decision-support systems is expected to accelerate the translation of PRLMs from research to clinical application, ultimately establishing them as a cornerstone technology for precision therapy and immune intervention in inflammatory diseases.

## Conclusions

Since the isolation of cortisone from the adrenal gland in 1934, steroid drugs have been the cornerstone of anti-inflammatory therapy, offering potent symptom control [317]. However, decades of clinical experience and mechanistic studies have revealed their inherent limitations, including broad immunosuppression, cumulative toxicity, and delayed tissue repair [318, 319]. Even targeted biologics, such as IL-1β monoclonal antibodies, whereas more precise, can compromise immune surveillance and increase susceptibility to infections and tumorigenesis. These shortcomings underscore the need for therapeutic strategies that go beyond suppression and align more closely with the endogenous mechanisms of resolution.

The discovery of PRLMs has reframed our understanding of inflammation. Inflammation can be actively resolved through a coordinated biological program rather than being passively silenced. PRLMs, as endogenous lipid-derived mediators, regulate immune cell phenotypic switching, phagocytosis, migration, and cytokine networks to drive the transition from inflammation to repair processes. Unlike conventional drugs, they restore homeostasis through “gentle intervention,” avoiding systemic immunosuppression. This unique property renders them particularly promising for chronic inflammatory disorders, metabolic syndromes, cardiovascular injuries, and NDDs that require long-term immune modulation. Recent advances, including the optimization of synthetic analogs and development of receptor-targeted agonists, further highlight their potential as master regulators of immune ecosystem reconstruction and tissue microenvironment reprogramming.

However, the clinical translation of PRLMs remains challenging. Their biosynthetic pathways are complex, unstable, and highly dynamic, and the lack of precise dosing windows and real-time monitoring technologies generates markedly uncertainty in therapeutic application [262]. Preclinical studies, such as MI models, illustrate the double-edged nature: early administration of RvE1 mitigates inflammatory damage, whereas delayed treatment impedes reparative macrophage recruitment and angiogenesis, ultimately delaying tissue regeneration. These observations emphasize that therapeutic efficacy is closely associated with the spatiotemporal dynamics of inflammation, underscoring the need for diagnostic and therapeutic frameworks grounded in individualization, temporality, and tissue specificity.

Future research should focus on the systematic integration of mechanistic decoding, drug engineering, and precision applications. Multi-omics technologies can be leveraged to construct dynamic metabolic atlases of PRLM–lipid pathways, enabling the identification of metabolic bottlenecks and therapeutic windows. Advances in delivery science, such as nanocarriers, self-assembling platforms, and biomimetic encapsulation systems, offer opportunities to enhance stability, tissue targeting, and pharmacological durability. Moreover, incorporating PRLM-mediated regulation into lifelong health management, from early screening of high-risk populations to postoperative recovery and chronic disease interventions, highlights their broader potential. With the continued integration of bioengineering, high-dimensional omics, and artificial intelligence, PRLMs are poised to become pivotal molecular hubs bridging immune homeostasis and tissue repair, opening new avenues for precision and systemic therapies for complex diseases.

## Data Availability

Not applicable.
